# Spontaneous Activity Predicts Survival of Developing Cortical Neurons

**DOI:** 10.3389/fcell.2022.937761

**Published:** 2022-08-10

**Authors:** Davide Warm, Davide Bassetti, Jonas Schroer, Heiko J. Luhmann, Anne Sinning

**Affiliations:** Institute of Physiology, University Medical Center of the Johannes Gutenberg University, Mainz, Germany

**Keywords:** development, spontaneous activity, cortical neurons, apoptosis, machine learning, MEA, calcium imaging

## Abstract

Spontaneous activity plays a crucial role in brain development by coordinating the integration of immature neurons into emerging cortical networks. High levels and complex patterns of spontaneous activity are generally associated with low rates of apoptosis in the cortex. However, whether spontaneous activity patterns directly encode for survival of individual cortical neurons during development remains an open question. Here, we longitudinally investigated spontaneous activity and apoptosis in developing cortical cultures, combining extracellular electrophysiology with calcium imaging. These experiments demonstrated that the early occurrence of calcium transients was strongly linked to neuronal survival. Silent neurons exhibited a higher probability of cell death, whereas high frequency spiking and burst behavior were almost exclusively detected in surviving neurons. In local neuronal clusters, activity of neighboring neurons exerted a pro-survival effect, whereas on the functional level, networks with a high modular topology were associated with lower cell death rates. Using machine learning algorithms, cell fate of individual neurons was predictable through the integration of spontaneous activity features. Our results indicate that high frequency spiking activity constrains apoptosis in single neurons through sustained calcium rises and thereby consolidates networks in which a high modular topology is reached during early development.

## 1 Introduction

Immature neuronal networks display several patterns of spontaneous activity, whose spatial and temporal organization plays an instrumental role for circuit assembly in the developing brain (for review, see Ben-Ari, 2001; Spitzer, 2006; Kirkby et al., 2013; Luhmann et al., 2016). By driving neuronal migration and the establishment of connectivity (Xu et al., 2011; Bando et al., 2016), activity patterns coordinate the integration of neurons into the cortex and distinctively shape cortical areas during early development (for review, see Kilb et al., 2011; Martini et al., 2021). Concomitantly, high levels and complex patterns of spontaneous activity across different brain regions are associated with low rates of apoptosis (Blanquie et al., 2017a; Denaxa et al., 2018; Duan et al., 2020) and disruption of adequate neuronal activity in the cortex determines an increase in cell death (Ikonomidou et al., 1999; Heck et al., 2008; Lebedeva et al., 2017). Indeed, besides genetic programs and neurotrophic factors, electrical activity and synaptic inputs directly regulate neuronal survival in the central nervous system (for review, see Dekkers et al., 2013). In turn, alterations of physiological apoptotic rates in critical phases of development are associated with abnormal structure of cortical circuits (Fang et al., 2014) and can lead to impaired functionality of the brain (Nakamura et al., 2016; for review, see Wong and Marín, 2019).

Spontaneous activity in the developing cortex evolves from uncorrelated neuronal firing to synchronized network oscillations in local neuronal assemblies (for review, see Egorov and Draguhn, 2013; Molnár et al., 2020). Here, synchronous network activity is marked by discontinuous high frequency oscillations, which are observed at regular intervals in several animal models, both *in vivo* and *in vitro* (Allène et al., 2008; Yang et al., 2009; for review, see Khazipov and Luhmann, 2006; Luhmann et al., 2022). At the cellular level, these rhythmic discharges translate into bursts of action potentials (Cunningham et al., 2004; Minlebaev et al., 2007), which are thought to strengthen synaptic contacts (Winnubst et al., 2015) and ensure communication between neurons (for review, see Lisman, 1997). High frequency firing leads to intracellular calcium transients (Murphy et al., 1992) that trigger numerous transcriptional programs (Tyssowski et al., 2018) and modulate key molecular processes for network development (Miyasaka and Yamamoto, 2021; for review, see Greer and Greenberg, 2008). Among these, burst activity and somatic calcium rises have been shown to support survival of developing neuronal populations (Collins et al., 1991; Wong Fong Sang et al., 2021), while lower calcium activity has been observed in neurons prior to cell death (Murase et al., 2011; Wong et al., 2018).

Hence, spontaneous activity guides the assembly of neuronal circuits, which are structurally characterized by densely interconnected neighboring neurons and long-range patchy connections between distant neuronal clusters (Voges et al., 2010; Robert et al., 2012). Such a modular topology fosters the emergence and sustainment of synchronous burst events (Okujeni et al., 2017) and has been demonstrated to be functionally efficient for information transfer and processing in cortical networks (for review, see Bullmore and Sporns, 2009). Although activity plays a predominant role in neuronal circuit formation and its silencing can dramatically affect network maturation (for review, see Ackman and Crair, 2014), absence of electrical activity does not lead to generalized cell death (Fishbein and Segal, 2007; Priya et al., 2018; Wong et al., 2018). Moreover, during early development, a large proportion of neurons is silent or only sparsely displays action potentials (Corlew et al., 2004; Sun et al., 2010). Therefore, the relationship between early spontaneous activity and survival of individual neurons is still elusive and the question whether activity profiles can anticipate survival or death of individual neurons remains open.

To unravel the relevance of spontaneous activity from single neuron to the network level for neuronal survival, we longitudinally followed developing neurons in primary cortical cultures. The transgenic expression of a genetically encoded calcium indicator and a fluorescent nuclear tag allowed the direct observation of single neurons while preserving stereotypical features of network development. By combining microelectrode array (MEA) electrophysiology with calcium imaging, we monitored spontaneous firing in large neuronal populations and investigated whether spiking frequency differentially encodes for survival of individual neurons. To characterize pro-survival factors of active, as well as silent neurons, we explored how both the local cellular surrounding and the emerging functional connectivity of the network influence cell death. Finally, we show that by means of machine learning algorithms, it is possible to predict neuronal survival based on activity profiles and infer activity features that are decisive for survival of developing cortical neurons.

## 2 Materials and Methods

### 2.1 Experimental Model and Techniques

#### 2.1.1 Primary Cortical Cultures

Primary neuronal cultures were prepared from newborn (postnatal day 0) C57BL/6NRj mice (RRID: MGI:6236253). All animal experiments conducted in this study were in accordance with national and European laws for the use of animals in research (2010/63/EU) and were approved by the local ethical committee (Landesuntersuchungsamt Rheinland-Pfalz 23.177–07/G10-1-010 and G20-1-006). After decapitation, brains were extracted from the skull and transferred to ice-cold Ca^2+^- and Mg^2+^-free Hank’s balanced salt solution (HBSS, Gibco, Thermo Fisher Scientific, Waltham, Massachusetts, United States) supplemented with penicillin and streptomycin (50 U/mL, Sigma-Aldrich, Merck, Darmstadt, Germany), sodium pyruvate (11 mg/mL, Sigma-Aldrich), glucose (0.1%, Sigma-Aldrich), and HEPES (10 mM, Sigma-Aldrich). Upon dissection of the cerebral hemispheres, meninges were removed and cortices isolated from the hippocampus, striatum and thalamic nuclei. Dissected cortices were incubated in 0.05% Trypsin-EDTA (Gibco) at 37°C for 20 min, followed by DNAse digestion (200 U/mL, Sigma-Aldrich) at room temperature (RT) for 5 min. Trypsinization was blocked by washing steps with HBSS followed by Minimal Essential Medium (Gibco) supplemented with 10% horse serum (Gibco) and 0.6% glucose. Cells were dissociated by mechanical trituration through fire-polished pipettes with declining diameter and filtered through a cell strainer with a mesh size of 40 µm (Greiner Bio-One, Frickenhausen, Germany). Alive cells were counted after trypan blue (Sigma-Aldrich) staining and seeded on microelectrode arrays (standard glass MEAs, NMI Technologie Transfer, Reutlingen, Germany), glass coverslips (NeuroClean, PrimeGlass, Forstinning, Germany) or 24 and 96 multiwell plate (Greiner Bio-One) with an initial plating density of approximately 2,000–2,500 cells/mm^2^. MEAs were coated with polyethyleneimine (0.05% in borate-buffered solution, Sigma-Aldrich) and coverslips or multiwell plates with poly-L-ornithine (0.1 mg/mL, Sigma-Aldrich). After 25 min, plating medium was suctioned and cells were incubated in Neurobasal™ A medium (Gibco) supplemented with 2% B27 (Gibco) and 1 mM L-glutamine (Gibco). Cells were maintained at 37°C in a humidified atmosphere (95% air and 5% CO_2_) and 30% of the culture medium was exchanged at day *in vitro* (DIV) 7 with BrainPhys™ medium (StemCell Technologies, Vancouver, Canada) supplemented with SM1 (StemCell Technologies). At DIV 2, neuronal cultures were treated with 5 µM ara-C (Sigma-Aldrich) to inhibit glial proliferation.

#### 2.1.2 Viral Transduction

Cortical neurons were transduced at DIV 1 with a recombinant adeno-associated virus (rAAV1/2, appr. 1–4 × 10^4^ viral genomes per cell) carrying GCaMP6s-P2A-nls-dTomato under the pan-neuronal promoter hSyn1. Plasmid DNA for generation of AAV-hSyn1-GCaMP6s-P2A-nls-dTomato was a gift from Jonathan Ting (Addgene plasmid # 51084). Viral production was done according to During et al. (2003). In brief, HEK293 cells were co-transfected with plasmids carrying the rep and capsid sequence of AAV serotype 1 and 2, a helper plasmid and the above-described plasmid carrying the genes of interest flanked by inverted terminal repeat (ITR) sequences. After 48 h, rAAV was harvested, extracted and purified via Heparin columns (HiTrap^®^ Heparin columns, Sigma-Aldrich). Virus titer was determined by quantitative real time PCR.

#### 2.1.3 MEA Recordings

Extracellular electrophysiological recordings were performed using planar 120-channel microelectrode arrays (120MEA100/30iR-ITO-pr, Multi Channel Systems, Harvard Bioscience, Inc., Holliston, Massachusetts, United States) with a MEA2100 system (Multi Channel Systems). Electrodes on MEAs had a layout of 12 × 12, a tip diameter of 30 µm and an inter-electrode (center to center) distance of 100 µm. Recordings were carried out for 10 min and temperature was maintained at 37°C with a temperature controller (TC02, Multi Channel Systems). Analog signals were amplified (bandwidth 0.1 Hz–10 kHz) and acquired with MC_Rack 4.6 software (Multi Channel Systems) at a sampling frequency of 50 kHz. Raw traces were processed with a 200 Hz high-pass filter (Butterworth second-order) and spikes were detected using a negative threshold-based detector set to 7× the standard deviation of the noise level. Only channels that recorded at least 1 spike per minute were considered active and included in the analysis. Mean spike rate was computed as the average number of spikes recorded across all channels per second.

#### 2.1.4 Live-Cell Imaging

Live-cell imaging was carried out with an upright microscope (BX61WI, Olympus, Tokyo, Japan) connected to a digital CCD camera (ORCA-R2, C10600-10B, Hamamatsu Photonics K.K., Hamamatsu, Japan) and a xenon arc light source (MT20-E, Olympus). Fluorescence and bright field images were taken with a 10× water-immersion objective (UMPLFLN10XW, Olympus) and acquired with xcellence software (Olympus). For calcium imaging, time-lapse videos were sampled at a frequency of 2 Hz with a green filter set (FITC, excitation 485/20, emission 521 nm). Red filter set (CY3, excitation 560/25 nm, emission 607 nm) was used for imaging of nls-dTomato or calcein red-orange signals. Electrophysiological and optical recordings were synchronized with TTL pulses delivered via a microcontroller board (Leonardo, Arduino, Boston, Massachusetts, United States).

#### 2.1.5 Cell Viability

Viability of primary neuronal cultures was assessed with alamarBlue™ HS Cell Viability Reagent (Invitrogen, Thermo Fisher Scientific), based on the redox indicator resazurin. Neurons were incubated with Alamar Blue (10% v/v in culturing medium) at 37°C in humidified 95% air and 5% CO_2_ for 60 min. Fluorescence was measured with a microplate reader (Infinite M1000, Tecan, Maennedorf, Switzerland) with excitation wavelength of 560 nm and emission of 590 nm. Background from blank wells was subtracted and all values were normalized to averaged untreated control.

Single cell viability was assessed using CellTrace™ Calcein Red-Orange AM (Invitrogen) or Hoechst (Invitrogen) according to the manufacturer’s guidelines. In short, cell cultures were incubated for 30 min at 37°C with calcein AM or Hoechst at a final concentration of 1 μM and 5 μg/mL, respectively. After subsequent wash with phosphate-buffered saline (PBS) to minimize background fluorescence, neuronal cultures were imaged in transparent culture medium.

#### 2.1.6 Caspase Assay

Caspase activity was measured by the luminescent Caspase-Glo^®^ 3/7 Assay (Promega, Madison, Wisconsin, United States). This homogeneous, luminescent assay provided a luminogenic caspase-3/7 substrate, which contained the tetrapeptide sequence DEVD, in a reagent optimized for caspase activity, luciferase activity, and cell lysis. The protocol was performed according to the manufacturer’s guidelines. In short, equal amounts of Caspase-Glo^®^ 3/7 reagent and PBS were added to the cells upon removal of the medium. Cells and buffer were mixed for 30 s using a plate shaker at 400 rpm (Infinite M1000, Tecan) and incubated for 60 min at RT. Luminescence was measured with a microplate reader (Infinite M1000, Tecan) and normalized for statistical analysis.

#### 2.1.7 Immunocytochemistry

For immunocytochemical analysis, cells were fixed in 4% formaldehyde in phosphate buffer (ROTI^®^ Histofix 4%, Carl Roth, Karlsruhe, Germany) for 15 min and washed with PBS. Unspecific binding of antibodies was blocked with normal donkey serum (Cat #017-000-121, Jackson ImmunoResearch, Laboratories Inc., Dianova Hamburg, Germany)/0.3% (v/v) triton (Triton^®^ X-100, Sigma-Aldrich) in PBS 0.01 M for 2 h at RT. For antigen detection, cells were incubated overnight at 4°C in PBS 0.01 M/2% bovine serum albumin (Cat #001-000-161, Jackson ImmunoResearch)/0.05% sodium azide (S002, Sigma-Aldrich)/0.1% triton with the following primary antibodies: mouse monoclonal (Cat #MAB377, Millipore, Merck, RRID: AB_2298772) or rabbit monoclonal (Cat #ab177487, Abcam, Cambridge, United Kingdom, RRID: AB_2532109) anti-NeuN, goat polyclonal anti-mCherry (Cat #AB0040-200, Sicgen, Lisbon, Portugal, RRID: AB_2333092), goat polyclonal (Cat #AB0020-200, Sicgen, RRID: AB_2333100) or rabbit polyclonal (Cat #A-11122, Life Technologies, Thermo Fisher Scientific, RRID: AB_221569) anti-GFP, rabbit polyclonal anti-GAD67 (Cat #198 013, Synaptic Systems, Goettingen, Germany, RRID: AB_2107718). For fluorescence labeling, the following fluorophore conjugated secondary antibodies were used: DyLight 405 – donkey anti-Rabbit (Cat #711-475-152, Jackson ImmunoResearch, RRID: AB_2340616), Cy2 – donkey anti-Goat (Cat #705-225-147, Jackson ImmunoResearch, RRID: AB_2307341), DyLight 488 – donkey anti-Rabbit (Cat #A120-208D2, Bethyl Laboratories, Biomol, Hamburg, Germany, RRID: AB_10627668), Cy3 – donkey anti-Goat (Cat #705-165-147, Jackson ImmunoResearch, RRID: AB_2307351) or Alexa Fluor 647 – donkey anti-Mouse (Cat #715-605-151, Jackson ImmunoResearch, RRID: AB_2340863), Alexa Fluor 647 – donkey anti-Rabbit (Cat #711-605-152, Jackson ImmunoResearch, RRID: AB_2492288). Images from fixed neurons were taken with a 10× objective (UPLFLN10X2PH, Olympus) with an epifluorescence microscope (IX81, Olympus) connected to a CCD camera (XM10, Olympus) using the cellSens software (Olympus). Representative images were taken with a confocal laser scanning microscope (TCS SP5, Leica Microsystems, Wetzlar, Germany) at a magnification of 40× with an oil-immersion objective (HC PL APO 40×/1,30 OIL PH3 CS2, Leica Microsystems) using LAS AF software (Leica Microsystems). Images were subsequently analyzed in Fiji. For quantification of expression levels, an automatic routine was written in ImageJ Macro language.

#### 2.1.8 Pharmacology

In a subset of experiments, staurosporine (1.5 µM, Sigma-Aldrich), a non-selective protein kinase inhibitor (Koh et al., 1995) was administered to induce apoptosis in cortical cultures. In another subset of experiments, tetrodotoxin (TTX, 1 μM, Tocris, Bio-Techne, Minneapolis, Minnesota, United States) and isradipine (Isr, 10 μM, Sigma-Aldrich) were used to prevent Na^+^ action potentials and block voltage-gated Ca^2+^ channels.

### 2.2 Data Analysis and Statistics

#### 2.2.1 Image Preprocessing

Image preprocessing was performed in Fiji (Schindelin et al., 2012) using a custom written routine. First uneven illumination due to spherical aberration was corrected according to a blurred mask obtained with *Gaussian Blur* filter. Multiple imaging sessions, including bright field (BF), nls-dTomato (CY3) and GCaMP6s (FITC) time-lapse images, were registered and aligned to the first experiment time-point. Electrodes and neuronal region of interests (ROIs) were obtained with *Analyze Particles* after image automatic thresholding. *Minimum* thresholding was applied to BF picture to identify dark electrodes. For neuron ROI identification, *Triangle* thresholding followed by *Watershed* segmentation was applied to CY3 nls-dTomato pictures. A background ROI devoid of cells and neurites and a reference ROI within it were delineated. The reference mean gray values and the background minimum value were extracted for normalization and offset correction of the calcium traces, respectively.

#### 2.2.2 Cell Identification and Cell Fate Detection

Single neuron identification was based on the thresholded nuclear nls-dTomato signal using a custom written ImageJ macro in Fiji. In detail, neurons were included for further analysis when their nuclei at DIV 9 had an area of at least 50 μm^2^ and a circularity of 0.5. Somatic areas were delimited fitting an ellipse to nuclear ROIs and enlarging them by a scaling factor of 1.5. Somatic ROIs were carefully evaluated across all time points to remove artifacts. After definition of ROIs, the following properties were extracted: area, centroid, and area fraction. Over the following time points (DIV 12 and 15), a neuron was considered as dead when the area fraction was ≤15%. Conversely, cells that displayed an area fraction >25% were considered alive. Additionally mean gray value from nls-dTomato raw images were extracted.

#### 2.2.3 Calcium Imaging Analysis

Raw calcium traces based on the somatic GCaMP6s signal were extracted from FITC time-lapse videos by computing the mean gray values of each neuron for every frame using the *Multi Measure* tool in Fiji. Raw calcium traces were then imported into Matlab 9.8 (The MathWorks Inc., Natick, Massachusetts, United States), normalized according to the reference trace, and the background intensity was subtracted. ΔF/F_0_ was computed using an open source toolbox (Romano et al., 2017) with detection of active periods (calcium transients) above a dynamic threshold (95% confidence interval). An additional static threshold (3× the standard deviation of the baseline noise σ) was applied. Finally, all traces were visually inspected to remove artifacts. Calcium peaks within identified calcium transients were detected using *findpeaks* function (Signal Processing Toolbox™, Matlab) using *MinPeakProminence* as name and *σ* as value argument. Neurons were defined as active when they displayed significant calcium elevations for at least 1 s per 5 min.

#### 2.2.4 Spike Sorting and Assignment to Optically Identified Neurons

Spikes were sorted with Offline Sorter (Plexon Inc., Dallas, Texas, United States) using two highly robust methods, K-means Scan (KMS) and Valley Seeking Scan (VSS) (Sukiban et al., 2019) with the following tunable parameters: for KMS a unit range of 1–7, whereas for VSS a parzen multiplier range of 0.5–1.5 with steps of 0.2. Sorted single units were assigned to optically identified neurons using a custom written GUI in Matlab. Each neuron was first assigned to its closest electrode tip within a maximum radius of 60 µm. For every active channel and close-by active neurons, sorted units and calcium traces were displayed. Sorted units were compared to unsorted spikes to confirm or merge over-sorted units into a single spike train. Finally, spike trains were assigned to neurons, after visual inspection of the presence of a peak in the cross-correlogram calculated between the calcium trace and spike timestamps binned in intervals of 0.5 s.

#### 2.2.5 Reconstruction of Spike Trains

Reconstructed spike timestamps were inferred from calcium signals using the MLspike toolbox (Deneux et al., 2016). MLspike algorithm relies on a physiological model that considers mainly three parameters: the unitary calcium response (amplitude), the decay time (tau) and the decay kinetic of the sensor (supra-linearity). The optimal parameters were chosen through a brute-force approach in which a parameter grid was tested on a subset of 45 neurons, which showed a clear match between calcium imaging and electrophysiological signals. For the amplitude, a range of 10 values expressed as a fraction between 0.05 and 0.5 of the max ΔF/F_0_ was used. Values below the standard deviation of the baseline noise σ were discarded. For tau, the selected values ranged between 0.5 and 2 s. For supra-linearity, polynomial fitting was chosen and values ranging from 0.1 to 0.9 were considered for p2 coefficient, while a constant value of 0.05 was used for p3 coefficient. Performance of each run was evaluated by estimating the error rate (ER) through the provided code between the recorded and the reconstructed spike trains. Best candidates for each cell were selected upon a grid search with the lowest 10% ER value as a performance metric. This procedure yielded for each parameter 90 candidates, among which the median was taken. Finally, the median of best parameters across all cells was taken, leading to the following values: 0.1 × max ΔF/F_0_ (amplitude), 0.7 s (tau), and 0.45 (p2).

#### 2.2.6 Firing Properties

For the characterization of single cell firing properties, we computed Ca^2+^ transient rate as the number of calcium transients per minute, Ca^2+^ peak rate as the number of calcium peaks per minute, spike rate as the number of spikes per second, on time as the total active period in minutes. Max spike frequency was calculated as the maximum number of reconstructed spikes per sampling interval 0.5 s or was set to 1 when the maximum inter-spike interval was ≥1 s. Bursts were detected using the Max Interval algorithm (Cotterill et al., 2016) with the following parameters: the maximum initial inter-spike interval to start the burst (0.1 s), the maximum inter-spike interval to define the burst end (0.6 s), the minimum interval between bursts (1.1 s), the minimum burst duration (0.5 s), and the minimum number of spikes in the burst (3). The following burst metrics were computed: burst rate as the mean number of burst per minute, spikes in burst as the number of spikes within bursts divided by the total number of spikes (%). Network firing parameters were computed as average values across all active neurons.

#### 2.2.7 Spatial Organization

Morphological clusters were identified using DBSCAN algorithm (Ester et al., 1996) over the neurons coordinates with the maximum distance between two points *Eps* set to 50 µm and minimum number of points *MinPts* of 1. Aggregation index was computed as:

$$Aggregation=\;\frac{1-\left(Clusters/Cells\right)}{\left(Cells-1\right)/Clusters}$$

where *Clusters* is the number of unique clusters found and *Cells* is the total number of cells in the network. The aggregation index can take any value between 0, when every cell is isolated, and 1, when all cells are counted within a single cluster. For each cell belonging to a cluster with size >1, respective neighbors were identified and the average and maximal values of their firing properties were computed.

#### 2.2.8 Functional Connectivity

Pairwise statistical dependence between reconstructed spike trains was estimated with the spike time tiling coefficient (STTC, Cutts and Eglen, 2014). The STTC method estimates the synchrony of two spike trains through the following formula:

$$STTC=\;\frac{1}{2}\;\left(\frac{P_{A}-T_{B}\;}{1\;-\;P_{A}\;T_{B}\;}\;+\;\frac{P_{B}-T_{A}\;}{1\;-\;P_{B}\;T_{A}\;}\right)$$

where PA is the proportion of spikes in channel A that occur within ± Δt of a spike from channel B and TB is the fraction of the total recording time that falls within ± Δt of a spike from channel B. PB and TA are calculated similarly. A time window of 0.5 s was chosen. By means of the STTC method, we computed connectivity matrices, in which pairwise correlations were comprised between +1 and −1, where +1 indicated autocorrelation and negative values anticorrelation.

Functional connectivity parameters were computed using the Brain Connectivity Toolbox (Rubinov and Sporns, 2010) in MATLAB 9.8 (The MathWorks Inc.). First, undirected connectivity networks were assembled by linking each pair of nodes where a positive correlation existed. The presence of silent cells led to the introduction of disconnected nodes and the generation of fragmented networks. Therefore, to measure topological properties of distance and path length, values of distance matrix were set to the total number of nodes and the harmonic mean of the shortest path length was used to compute the characteristic path length (for review, see Fornito et al., 2016). The following nodes parameters were extracted: node degree, mean shortest path length, betweenness centrality, clustering coefficient, local efficiency, and hubness. Hubness score was defined based on the definition by Van den Heuvel et al. (2009). At network level, the following parameters were extracted: connection density, characteristic path length, betweenness centrality, clustering coefficient, global efficiency, modularity and small-worldness. Small-worldness was based on the definition by Humphries and Gurney (2008).

#### 2.2.9 Machine Learning

Supervised classification of neurons according to cell fate (surviving or dying) was performed in Python 3.7. Measures from 1874 cells at DIV 9 describing firing properties, spatial organization and functional connectivity at single cell and network level were pooled and z-scored. Class labels were created based on the fate of the cell at the subsequent experimental session, i.e., DIV 12 (surviving or dying). To deal with the asymmetric distribution of surviving vs. dying cells in the dataset, we selected the Random Undersampling Boosting (RUSBoost) classifier and Balanced Random Forest Classifier from the imbalanced-learn python package (Lemaître et al., 2017). As a control, we trained the Dummy classifier from the scikit-learn library (Pedregosa et al., 2011), using the class priors. Feature selection was performed after hierarchical clustering, using Spearman’s rank correlation coefficient as a distance measure, and manually selecting representative variables from each group of parameters identified using a cut-off value of 0.5 based on the shortest Euclidean distance. The predictors included are summed up in Supplementary Figure S7.

In order to select the most suitable parameters, nested cross-validation was employed, using 10 inner folds and 10 outer folds, using stratification. The following parameter grid was considered: for the RUSBoost classifier, *n_estimators* = [100, 1000, 2000] and *learning_rate* = [0.05, 0.5, 1], and for the Random Forest classifier, *n_estimators* = [1000, 2000], *max_features* = [2, 5], *max_depth* = [50, 100, 150] and *min_samples_leaf* = [3, 4, 8].

The use of a Random Forest classifier provides the benefit of a simple way to evaluate how relevant is each variable for the performed prediction. Random Forest classifiers are composed by a collection of decision trees. Each tree is trained to find the best split of the dataset, and in each node of the tree, a feature is selected such that it maximizes the decrease in impurity. The feature importance values correspond to the normalized measure of how strong is, on average, the decrease in impurity for each feature. The removal of highly correlated variables allows a better interpretability of the Random Forest feature importance scores, because such correlation can lead to over/underestimation of individual variables in terms of contribution to the decision (Nicodemus et al., 2010). Feature importance was collected using an additional cross-validation round (10-folds).

In order to obtain a descriptive activity profile of cells that would be classified as dying or surviving, we selected all the cells that were more confidently predicted by the classifier in each class (i.e., >80% probability of assignment to a class, calculated using the *predict_proba* method) and calculated the median value for each feature.

#### 2.2.10 Statistics

All statistical tests were performed using GraphPad Prism 9.3 (GraphPad, La Jolla, California, United States). Normality of sample distributions was tested with Shapiro-Wilk test. Direct comparisons between two groups were performed with Student unpaired *t*-test for normally distributed data or nonparametric Mann-Whitney test when the samples were not following a Gaussian distribution. Two-samples Kolmogorov-Smirnov test was used to compare cumulative frequency distributions. Multiple groups were compared using either one-way ANOVA followed by Tukey’s multiple comparison post-hoc test, or Kruskal-Wallis test followed by Dunn’s or Dunnett’s multiple comparison post-hoc test, depending on whether the normality test was passed. Two-way ANOVA followed by Šidák’s multiple comparison was used to quantify the interaction between different factors. Linear dependency between two variables was evaluated with an F test. Chi-square test was used for testing independence of categorical variables and post-hoc pairwise comparisons were performed with Bonferroni correction to control for familywise error rate. Significance was considered at *p* values < 0.05. Data in bar charts are shown as mean ± standard deviation (SD), whereas box plots representing median and interquartile range (IQR) are shown with min–max whiskers.

## 3 Results

### 3.1 Network Activity Restricts Single Neuron Probability of Cell Death

**FIGURE 1:**
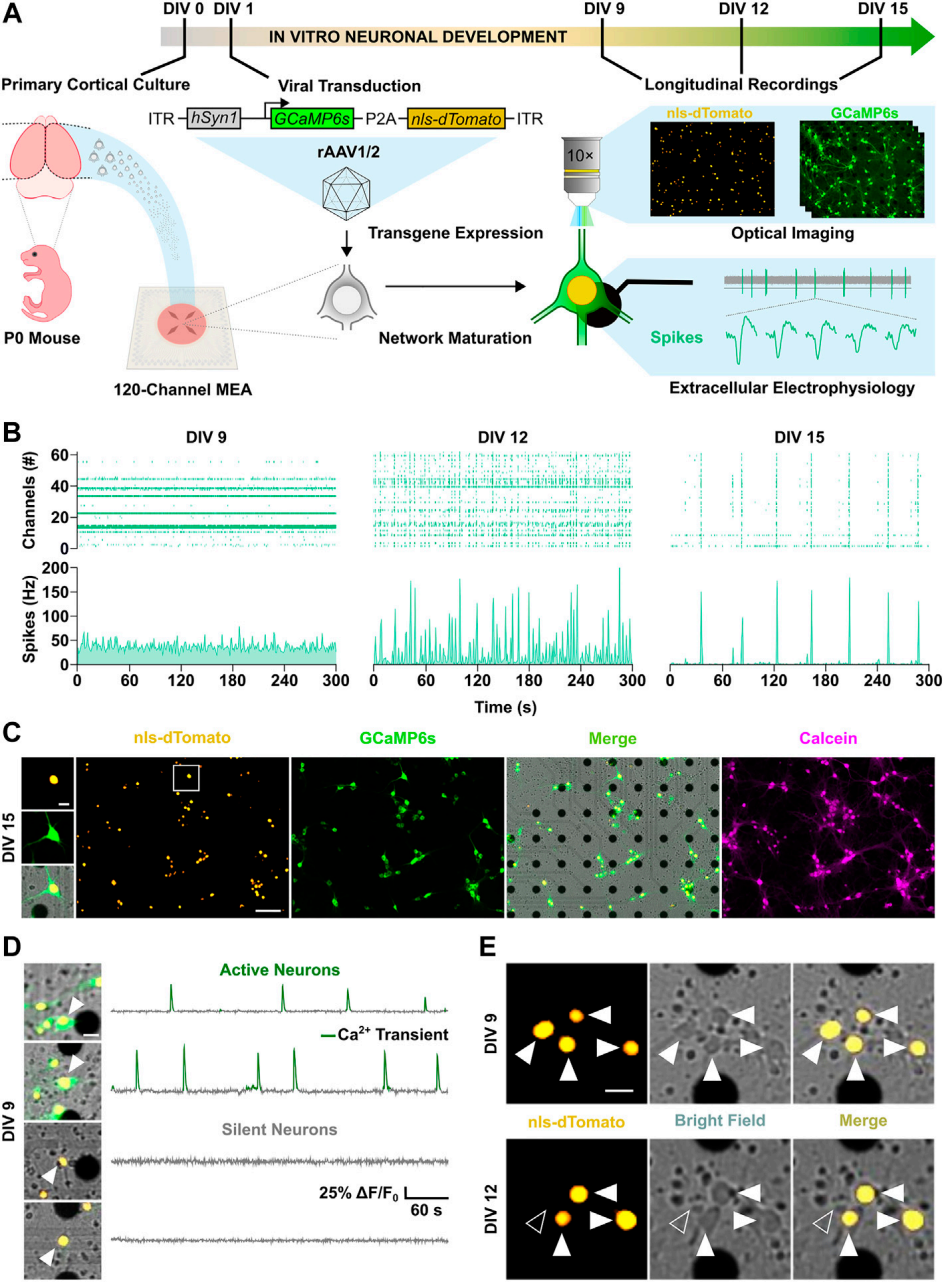
Spatiotemporal investigation of neuronal activity and apoptosis in developing cortical cultures. **(A)** Schematic overview of the experimental design. **(B)** Longitudinal electrophysiological MEA recordings over the second week *in vitro*. Spike raster plots (top panels) of active channels across development are shown together with corresponding spike count histograms (bottom panels). **(C)** Live-cell imaging of nuclear tag nls-dTomato (yellow) and maximal projection of calcium indicator GCaMP6s signal (green) in cortical neurons cultured on a MEA. Merge (yellow-green) image with the bright field channel highlights dark electrode tips. Post-hoc staining with calcein-AM dye (magenta) confirmed viability of identified neurons throughout the experiment. **(D)** Representative merge images and fluorescence traces of active (top) and silent neurons (bottom). Detected calcium transients are overlaid in green. **(E)** Longitudinal imaging of nuclear nls-dTomato signal allowed unbiased identification of neurons and reliable cell fate monitoring throughout the experimental time course. Surviving neurons are indicated by white arrowheads, whereas neurons with an apoptotic cell fate are marked by unfilled arrowheads. Scale bars represent 100 µm in overview images **(C)** and 20 µm in cropped images of 100 × 100 µm **(C–E)**.

Developing cortical networks are affected by higher rates of apoptosis when lower levels of activity are present (Ikonomidou et al., 1999; Heck et al., 2008; for review, see Blanquie et al., 2017b). It is however unclear whether the large proportion of silent neurons in early development encompasses the fraction of neurons with an apoptotic fate, and what relationship exists between neuronal activity and cell fate of individual neurons. For the spatiotemporal characterization of activity and apoptosis during network development, we used cortical cultures grown on microelectrode arrays (MEAs). To investigate if distinct activity features characterize surviving or dying neurons at a single cell level, neurons were transduced with a recombinant adeno-associated virus endowing them with two fluorescent reporters: the orange fluorescent nuclear tag nls-dTomato and the green fluorescent calcium indicator GCaMP6s (Figure 1A and Supplementary Figure S1A). With this strategy, about 80% of neurons in culture were targeted with a preferential tropism for glutamatergic neurons (Supplementary Figure S1B). Already after 9 days *in vitro* (DIVs), a cell fraction above 50% showed a marked expression of the transgenes (Supplementary Figure S1C). From this point onwards, we performed longitudinal experiments combining extracellular electrophysiological MEA recordings with live-cell imaging at 3-days intervals until DIV 15 (Figure 1B and Supplementary Videos S1–S3). The nuclear tag nls-dTomato allowed the identification and follow-up of neurons on the MEA throughout the experimental time course (Figure 1C), whereas the co-expression of GCaMP6s enabled to distinguish between active, i.e. displaying calcium transients, and silent neurons (Figure 1D). Importantly, cell death could be promptly detected by loss of the nls-dTomato signal upon nuclear membrane disruption triggered by the apoptotic program execution (Figure 1E). As a positive control, we confirmed that neuronal apoptosis, induced by the protein kinase inhibitor staurosporine (Koh et al., 1995), led to the loss of nuclear nls-dTomato signal within the expected time frame of about 3 h (Supplementary Figure S1D).

**FIGURE 2:**
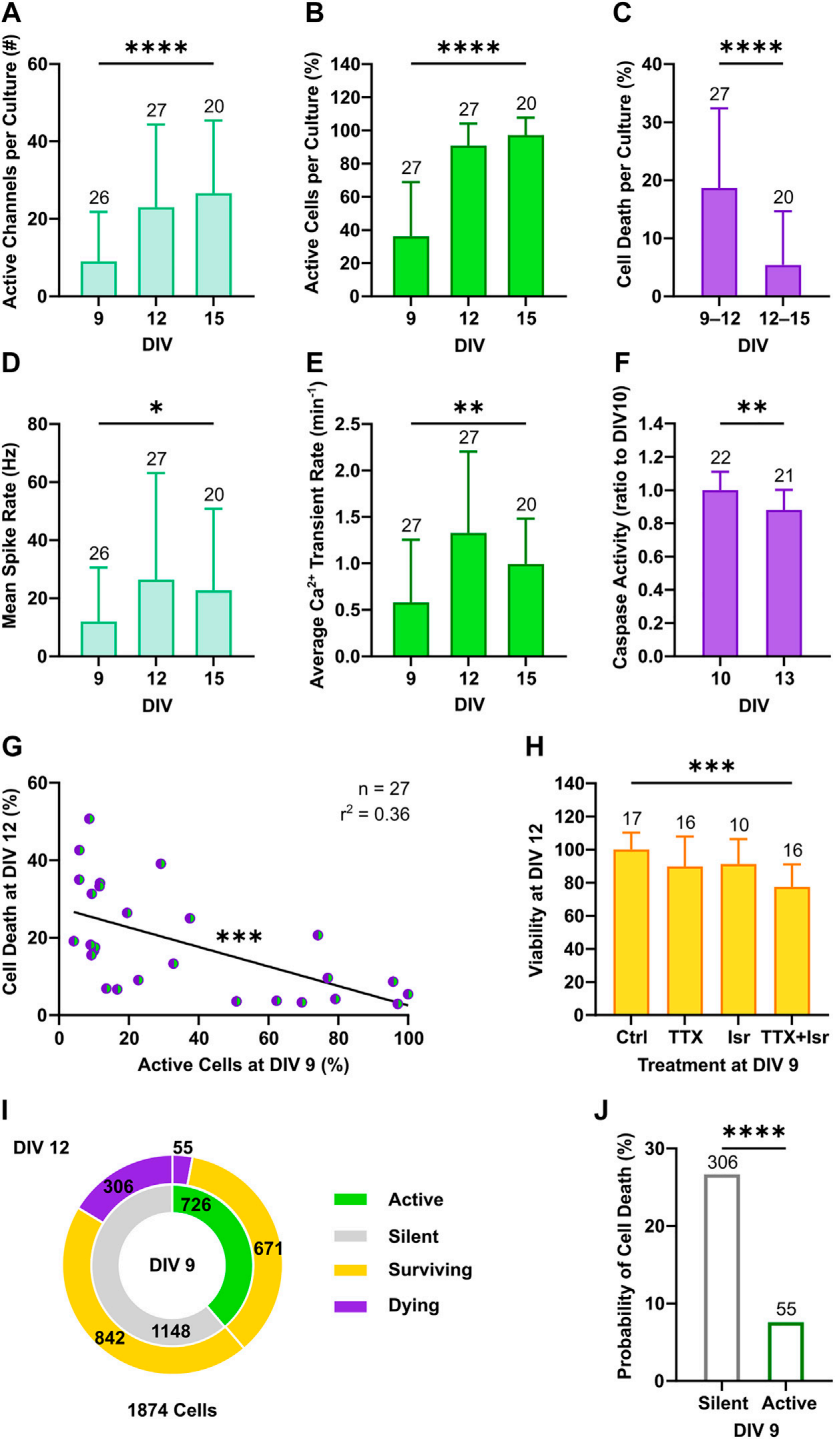
Neuronal activity decreases the probability of programmed cell death. **(A–C)** The number of active channels and the relative proportion of active neurons increased with development (Kruskal-Wallis test), whereas the percentage of cell death detected through longitudinal nls-dTomato imaging decreased from DIV 9–12 to DIV 12–15 (Mann-Whitney test). **(D,E)** The mean spike rate per culture recorded with MEA and the average rate of detected calcium transients increased from DIV 9 to DIV 12 and then stabilized (Kruskal-Wallis test). **(F)** In line with the observed rates of cell death, caspase activity in cultures was higher at DIV 10 compared to DIV 13 (Student unpaired *t*-test). **(G)** The proportion of active neurons in cultures at DIV 9 negatively correlated with the subsequent cell death rate at DIV 12 (F test). **(H)** Viability of culture at DIV 12 was reduced by combined application of tetrodotoxin (TTX) and isradipine (Isr) (Kruskal-Wallis test, followed by Dunnett’s multiple comparisons test, *p* < 0.0001), whereas application of TTX only led to a slight and non-significant reduction in viability. **(I)** The relative proportion of neurons with a survival cell fate was higher among active neurons as compared to the silent population. **(J)** On the single neuron level, the probability of cell death was higher in neurons, which were silent at DIV 9 as compared to active neurons (Chi-square test). Bar charts with mean ± SD or scatter plots with line indicating simple linear regression are shown. *p < 0.05, **p < 0.01, ***p < 0.001 and ****p < 0.0001.

Cortical neurons in culture self-organize into neuronal networks, in which wiring and pruning are accompanied by the progressive emergence and refinement of neuronal activity over the second week *in vitro* (Wagenaar et al., 2006; Sun et al., 2010). Consistently, a developmental increase in neuronal firing was captured between DIV 9 and 15 by both electrical and calcium activity measurements as shown by the increment in the number of active channel (Figure 2A, from 9 ± 12.8 to 26.7 ± 18.8, *p* < 0.001, Dunn’s multiple comparisons test) and in the relative proportion of active neurons within the network (Figure 2B, from 36.1 ± 32.9 to 97.3 ± 10.5, *p* < 0.0001, Dunn’s multiple comparisons test). In parallel, network firing rates steeply increased between DIV 9 and 12, as the mean spike rate shifted from 11.9 ± 18.6 to 26.4 ± 36.7 Hz (Figure 2D, *p* < 0.05, Dunn’s multiple comparisons test) and the average number of calcium transients per minute increased from 0.58 ± 0.68 to 1.33 ± 0.88 (Figure 2E, *p* < 0.001, Dunn’s multiple comparisons test). From DIV 12 to 15, mean rate of both spikes and calcium transients settled to 22.7 ± 28.1 Hz (Figure 2D, *p* > 0.99, Dunn’s multiple comparisons test) and 0.99 ± 0.49 transients per minute (Figure 2E, *p* = 0.92, Dunn’s multiple comparisons test), respectively. Between DIV 9 and 12, when neuronal network activity was still low and emerging, on average 18.6% ± 13.8 of neurons per culture underwent programmed cell death, whereas a significantly smaller fraction of 5.4% ± 9.2 died between DIV 12 and 15 (Figure 2C, *p* < 0.0001, Mann-Whitney test). In line with the higher rate of programmed cell death at early stage, activation of caspases, the main down-stream effectors in the intrinsic apoptotic cascade (Yuan and Yankner, 2000), was higher at DIV 10 compared to DIV 13 (Figure 2F, 1 ± 0.11 vs. 0.88 ± 0.12, *p* < 0.01, Student unpaired *t*-test).

Based on these findings, we focused our attention on the 3-days interval from DIV 9 to DIV 12 and correlated network activity features at DIV 9 with the subsequent rates of cell death at DIV 12. We observed a negative correlation between the proportion of active neurons at DIV 9 and cell death rates at DIV 12 across cultures (Figure 2G, F_(1, 25)_ = 14.19, *p* < 0.001). A similar linear dependency was present when plotting cell death rate as a function of the average calcium transient rate (Supplementary Figure S2A). Interestingly, this negative association between activity and cell death rate in cultures was not statistically significant when electrophysiological parameter such as number of active channels (Supplementary Figure S2B) and mean spike rate (Supplementary Figure S2C) were considered. The lack of correlation between electrical network parameters with cell death rates might reflect the fact that neuronal activity and cell survival were assessed in overlapping but not identical neuronal subpopulations or could point to a particular role of calcium signaling for the regulation of activity-dependent cell death (Turner et al., 2007). The latter hypothesis is supported by the observation that only combined blockade of voltage-gated Na^+^ and Ca^2+^ channels by tetrodotoxin (TTX, 1 µM) and isradipine (Isr, 10 µM) at DIV 9 resulted in a strong and significant decrease of neuronal survival at DIV 12 compared to control cultures (Figure 2H, 77.5 ± 13.6% vs. 100.1 ± 10.2%, *p* < 0.0001, Dunnett’s multiple comparisons test), while solely blocking action potentials with TTX only slightly and non-significantly reduced viability of neurons (Figure 2H, 89.9 ± 18% vs. 100.1 ± 10.2%, *p* = 0.12, Dunnett’s multiple comparisons test).

To investigate if also on a single neuron level the early activity profile was associated with subsequent survival or cell death, we pooled data from 1874 neurons imaged in 27 independent cultures, classified them according to the presence of calcium events at DIV 9 (active or silent) and correlated this with the respective cell fate observed at DIV 12 (surviving or dying) (Figure 2I). This analysis showed that the proportion of neurons with an apoptotic fate was greater among the silent subpopulation than the active one (Figure 2J, 26.7% vs. 7.6%, χ^2^ = 104.1, df = 1, *p* < 0.0001). Thus, the largest portion of apoptotic neurons was constituted by silent neurons albeit a minority of the active population also died.

In conclusion, the combination of longitudinal MEA recordings with optical imaging allowed to recapitulate the typical electrophysiological development of cortical networks *in vitro*, while enabling a detailed characterization of activity-dependent apoptosis of single neurons. The results suggest that the presence of activity at an immature developmental stage decreases the probability of death at the single neuron level.

### 3.2 Individual High Firing Rates Promote Neuron Survival

**FIGURE 3:**
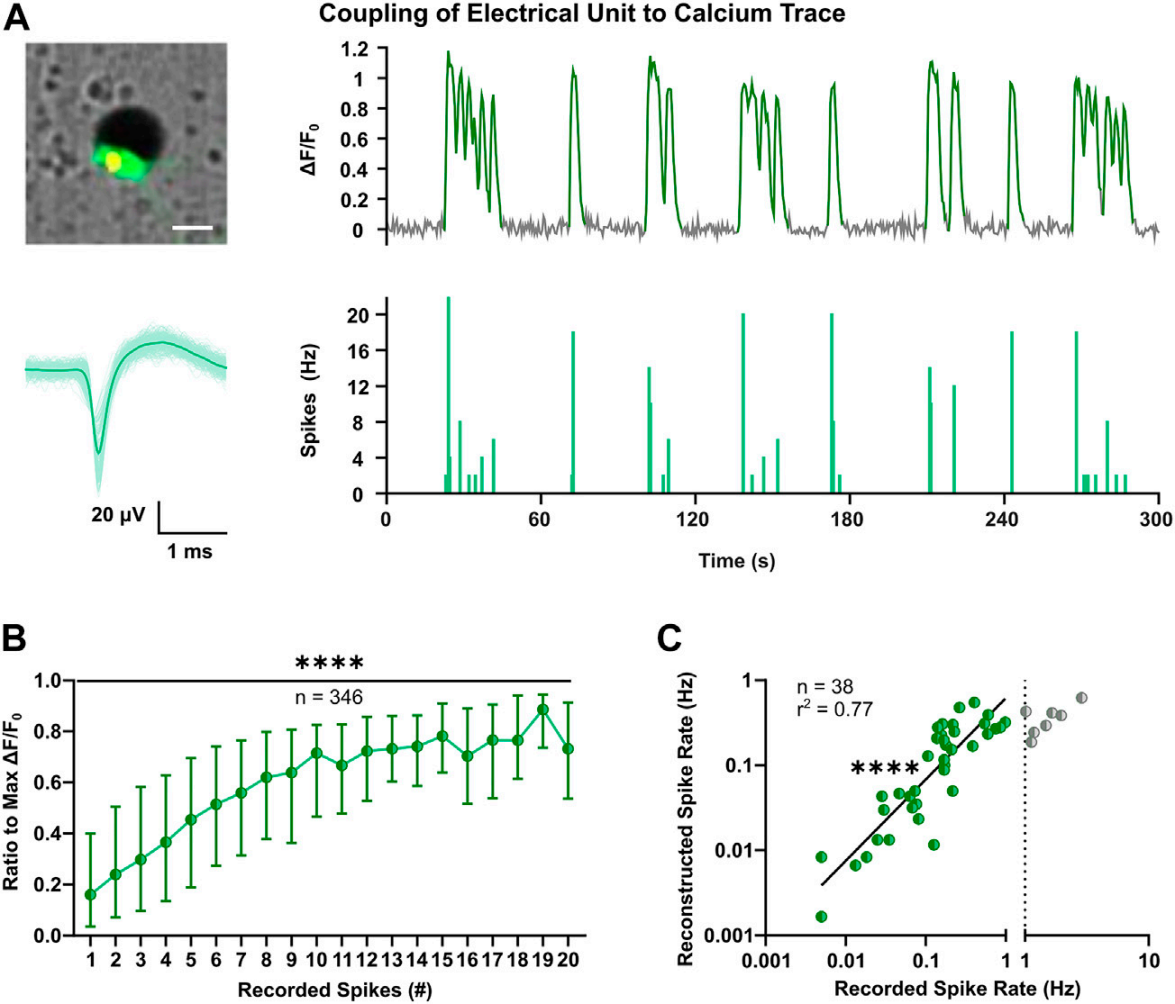
Optimization of spike inference based on coupling of extracellular voltage signals to intracellular calcium transients. **(A)** Representative example of coupling of the calcium trace (top-right) of a single neuron close to an electrode (top-left) and the corresponding electrophysiological signals. Superimposed waveforms and their mean (solid line) are shown on the bottom-left, whereas on the bottom-right the respective spike count histogram with bin size of 0.5 s is shown. Image scale bar represents 20 µm. **(B)** Comparison between electrically recorded spikes and GCaMP6s fluorescence, indicated as ratio to the max ΔF/F_0_ (Kruskal-Wallis test). Median and IQR are shown. **(C)** Correlation between mean spike rates recorded with MEA and inferred through MLspike algorithm with fitted parameters (F test). Values are log-transformed and axis show anti-log scale. Line indicates simple linear regression. ****p < 0.0001.

Cortical neurons during early development exhibit low-amplitude and sparse action potentials (Weir et al., 2015; for review, see Moody and Bosma, 2005), which can attenuate sensitivity of extracellular recordings and lower the performance of spike sorting (for review, see Buzsáki, 2004; Pedreira et al., 2012). To determine firing properties of all optically identified neurons, we applied spike inference on calcium traces using the MLspike toolbox (Deneux et al., 2016). The latter derives discrete spike timestamps from continuous fluorescence signals and was shown to have a good overall performance compared to other methods (Berens et al., 2018). The outcome of MLspike was benchmarked taking advantage of a subset of neurons in the close proximity of an electrode, to which both calcium traces and electrical signals could be clearly assigned (Figure 3A). To account for potential differences in transgene expression level (Supplementary Figure S3A), individual calcium responses were considered as ratios of the max ΔF/F_0_. This conversion enabled to infer a consistent correlation between the number of spikes recorded in 0.5 s bins and the respective calcium elevations in a large number of neurons (Figure 3B, *p* < 0.0001, Kruskal-Wallis test). The MLspike algorithm is based on a biophysical model that takes into account the unitary calcium response (amplitude) and kinetic properties of the calcium indicator (decay time and supra-linearity). To optimize the model parameters, we thus performed a grid search across different values and selected the best candidates based on the least error rates estimated between the recorded and reconstructed timestamps (Supplementary Figure S3B). Median values across each cell’s best reconstruction were taken for spike reconstruction (Supplementary Figure S3C–E). Resulting average rates of reconstructed spike timestamps were in good agreement with MEA-recorded firing rates up to 1 Hz (Figure 3C, F_(1, 36)_ = 120.3, *p* < 0.0001).

**FIGURE 4:**
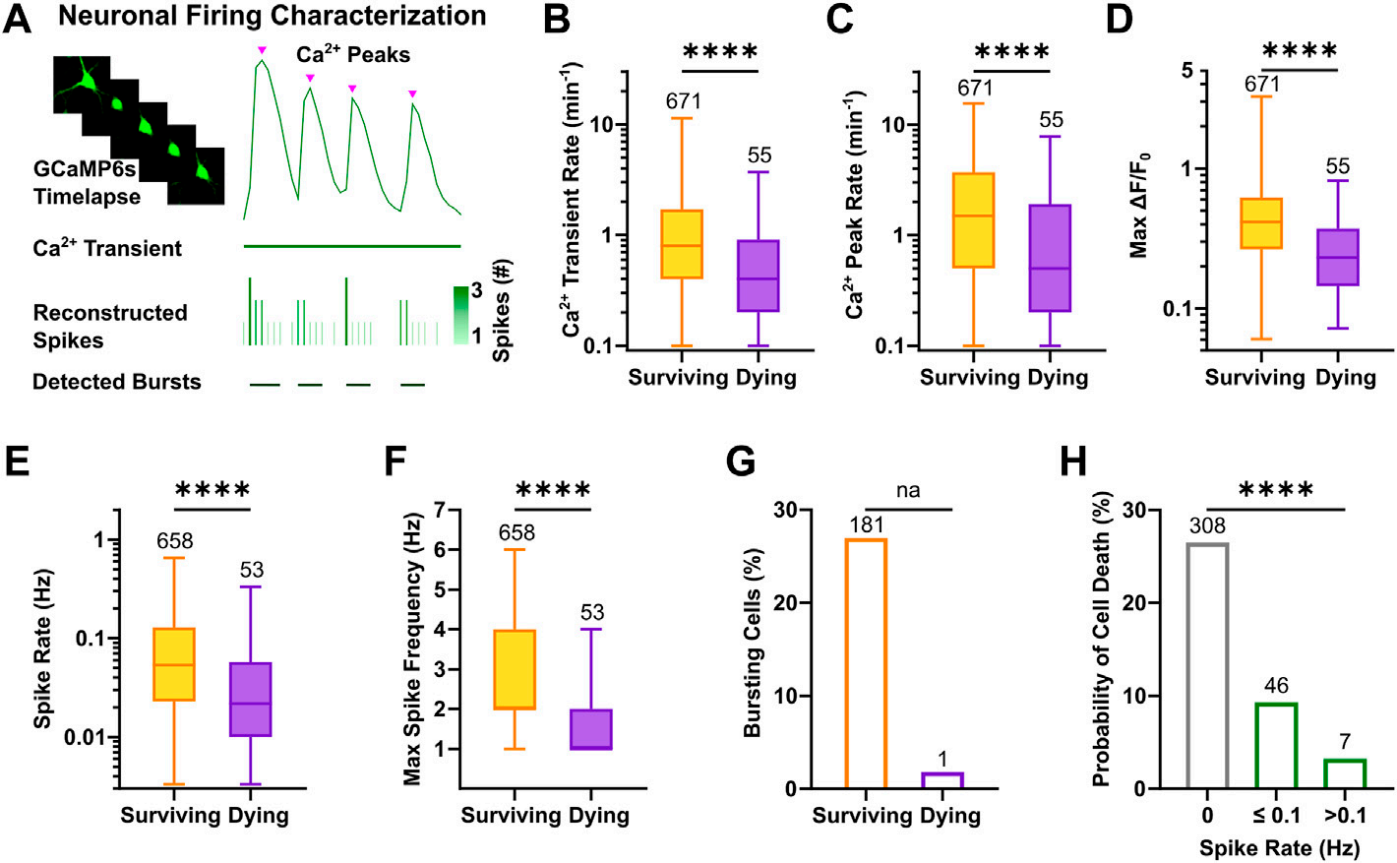
High frequency firing patterns promote neuronal survival. **(A)** Schematic overview of single neuron firing properties. **(B–D)** Rate of calcium transients and peaks as well as the maximum ΔF/F_0_ at DIV 9 were higher in neurons with a prospective survival fate (Mann-Whitney test). **(E,F)** Reconstructed spike rate and maximum spike frequency were higher in active neurons, which survived (Mann-Whitney test). **(G)** Bursts were almost exclusively detected in surviving neurons. **(H)** Probability of cell death was negatively correlated with spike rate of individual neurons (Chi-square test). Box plots with median and IQR and min–max whiskers are shown. **** *p* < 0.0001.

To characterize activity features of active cells that would subsequently undergo apoptosis or survive, calcium peaks were extracted from the displayed calcium transients, and reconstructed spike timestamps were analyzed to determine firing rates and bursting behavior (Figure 4A). The results showed that neurons, which were still viable at the next recording session at DIV 12, displayed higher calcium transient rates at DIV 9 (Figure 4B, 0.8, 0.4–1.7 vs. 0.4, 0.2–0.9, *p* < 0.0001, Mann-Whitney test) and a higher number of calcium peaks per minute compared to neurons that subsequently died (Figure 4C, 1.5, 0.5–3.7 vs. 0.5, 0.2–1.9, *p* < 0.0001, Mann-Whitney test). Coherently, the relative time in which surviving neurons displayed activity during the recording period was longer compared to neurons that died until DIV 12 (Supplementary Figure S4A). Importantly, not only the frequency but also the normalized amplitude of calcium events was larger in surviving neurons as indicated by the comparison of max ΔF/F_0_ (Figure 4D, 0.42, 0.27–0.62 vs. 0.23, 0.14–0.37, *p* < 0.0001, Mann-Whitney test). This difference was not due to variability in expression levels (Supplementary Figure S4B) or in the baseline calcium fluorescence (F_0_) across groups (Supplementary Figure S4C). Classification of active vs. inactive neurons based on reconstructed spikes almost completely confirmed the prior identification of activity in neurons (n = 711 based on reconstructed spiking vs. n = 726 neurons based on the calcium signals, i.e., 98% overlap). The systematic comparison of reconstructed firing parameters between active neurons with a prospective survival or dying cell fate showed that mean spike rates were significantly higher in surviving neurons (Figure 4E, 5.3 × 10^−2^, 2.3 × 10^−2^–1.3 × 10^−1^ vs. 2.2 × 10^−2^, 1 × 10^−2^–5.8 × 10^−2^, *p* < 0.0001, Mann-Whitney test). Also, the maximum frequency of spikes calculated as the instantaneous rate (i.e., number of spikes/sampling interval) was higher in surviving neurons (Figure 4F, 2, 2–4 vs. 1, 1–2, *p* < 0.0001, Mann-Whitney test). Interestingly, bursting behavior was an almost exclusive characteristic of surviving neurons (Figure 4G), which in median displayed 0.2 bursts per minute (Supplementary Figure S4D), and 25% of their spikes belonged to bursts (Supplementary Figure S4E). Of note, consistency between calcium and spike parameters was also maintained at network level (Supplementary Figure S4F–J). Classification of neurons according to their firing rate, outlined a higher probability of cell death for silent (Figure 4H, 26.5%, χ^2^ = 106.3, df = 2, *p* < 0.0001) or sparsely firing (9.3%) neurons compared to neurons showing at least 0.1 spikes per second (3.2%).

In conclusion, by fitting model parameters of an established spike inference method, we could reconstruct firing properties of all individual neurons. The comparative analysis of intrinsic activity features suggests that large amplitude of calcium responses, high frequency firing and the presence of bursting activity are beneficial for survival of neurons during early development in neuronal networks.

### 3.3 Neighbors’ Activity is a Protective Factor Against Cell Death

**FIGURE 5:**
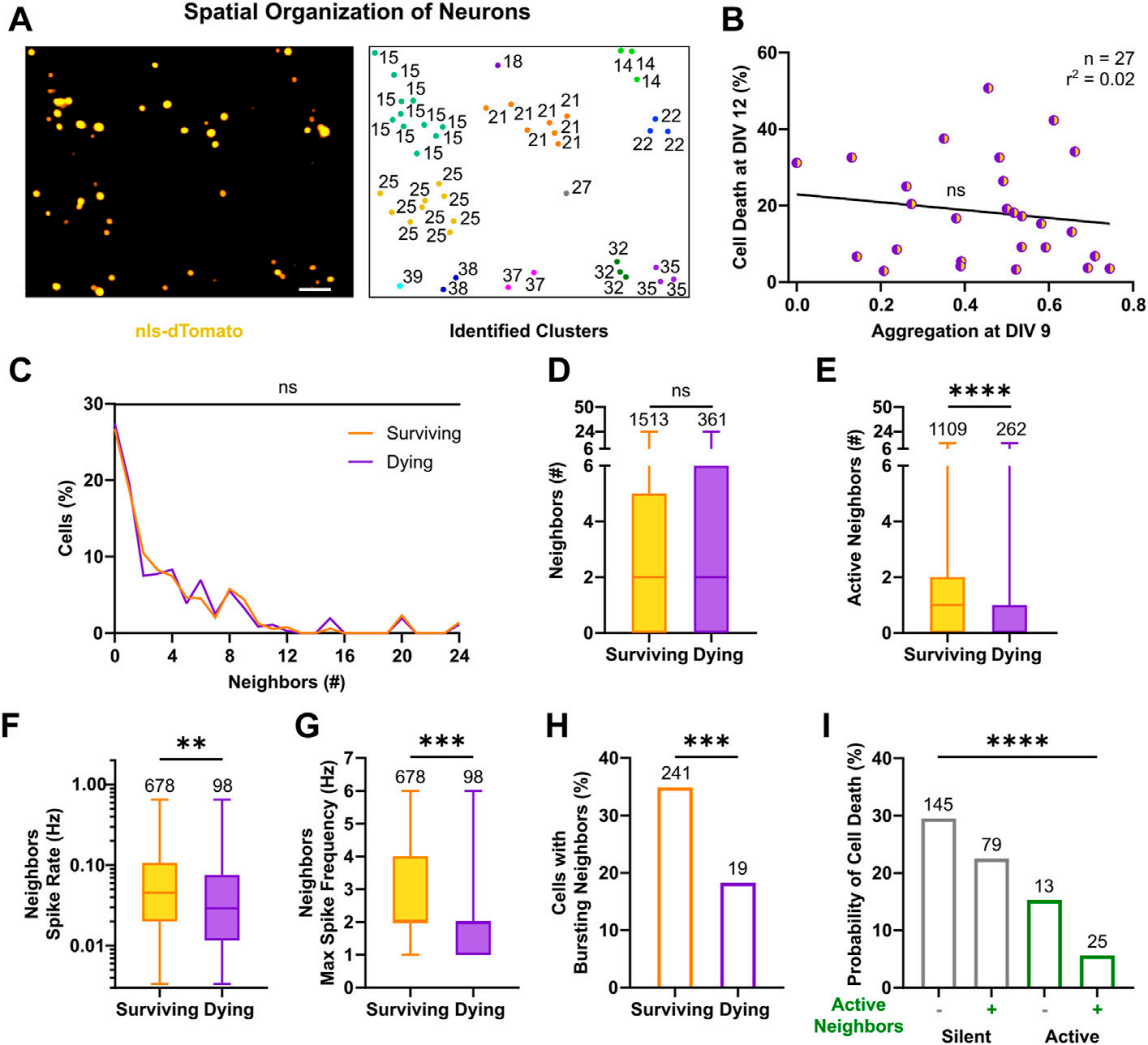
Activity of neighbors in neuronal clusters exerts a pro-survival effect. **(A)** Identification of neuronal clusters based on nls-dTomato signal in a representative field of view (left). Neurons are depicted based on their physical positions with respective cluster number (right). Image scale bar represents 50 µm. **(B)** Cell death rates at DIV 12 were not correlated with respective aggregation indices of cultures at DIV 9 (F test). Line indicates simple linear regression. **(C,D)** No significant difference in the number of nls-dTomato-positive neurons in physical proximity (i.e., neighbors <50 µm) was present between surviving and dying neurons, neither in the relative distributions (Kolmogorov-Smirnov test) nor in the median values (Mann-Whitney test). **(E–G)** The number of identified active neighbors, the average and maximum spike rates of neighbors were higher in neurons, which survived (Mann-Whitney test). **(H)** The relative percentage of active neighbors, which displayed bursting patterns, was higher for surviving neurons as compared to neurons with a prospective cell death fate (Chi‐square test). **(I)** For silent and active neurons, the presence of active neighbors in their vicinity decreased their probability of cell death (Chi‐square test). Box plots with median and IQR and min–max whiskers are shown and **p < 0.01, ***p < 0.001 and ****p < 0.0001.

Over the first days in culture, neurons relocate from randomly seeded positions into dense clusters of cells (Robert et al., 2012) before they start to form diffused synaptic connections and display spontaneous electrical activity (Shein Idelson et al., 2010). In order to assess whether the physical arrangement of neurons within local assemblies had any effect on neuronal survival or death, we analyzed the spatial organization of neuronal networks and the relative position of single neurons within these assemblies. For this purpose, we identified clusters by using the DBSCAN algorithm (Ester et al., 1996) with a radius of 50 µm (Figure 5A). In line with previous results (Robert et al., 2012), we found that cells display a tendency to form physical clusters, as they distributed preferentially within a 50 µm distance from their neighbors (Supplementary Figure S5A). At first, we investigated whether the sparseness of the network influenced overall survival or cell death rates. Therefore, we correlated densities and levels of aggregation with cell death rates of cultures. Neither the degree of aggregation (Figure 5B, F_(1, 25)_ = 0.56, *p* = 0.46) nor the mere density (Supplementary Figure S5B) of a culture was associated with significant changes in cell death rates. Next, we quantified, for each neuron, the number of neighbors and compared their distribution among the populations of surviving and dying neurons. The results showed, that regardless of their prospective cell fate (Figure 5C, *p* > 0.99, Kolmogorov-Smirnov test) most neurons had at least one neighbor (in total 73.2%) and in median every neuron had 2 neighbors (Figure 5D, 0–5 vs. 0–6, *p* = 0.98, Mann-Whitney test). Thus, the spatial organization of cortical networks did not correlate with neuronal death rates and the physical location of dying and surviving neurons within the network did not substantially differ.

We then investigated the activity profiles of neighbors. Interestingly, surviving neurons were surrounded by a higher number of active neighbors compared to dying neurons (Figure 5E, 1, 0–2 vs. 0, 0–1, *p* < 0.0001, Mann-Whitney test). Moreover, the proportion of active neighbors close to surviving cells was significantly higher than in the vicinity of dying neurons (Supplementary Figure S5C). Average spike rates across neighbors of surviving neurons were also significantly higher than those of neurons in the vicinity of dying neurons (Figure 5F, 4.6 × 10^−2^, 2 × 10^−2^–1.1 × 10^−1^ vs. 2.9 × 10^−2^, 1.2 × 10^−2^–7.5 × 10^−2^, *p* < 0.01, Mann-Whitney test). Furthermore, the maximal spike frequency among neighbors was higher for surviving neurons as compared to prospective dying neurons (Figure 5G, 2, 2–4, vs. 2,1–2, *p* < 0.001, Mann-Whitney test). Accordingly, bursting behavior was more often detected in neighbors surrounding surviving than dying neurons (Figure 5H, 34.9% vs. 18.3%, χ^2^ = 11.33, df = 1, *p* < 0.001), although its descriptive metrics, failed to capture any statistical significant difference (Supplementary Figures S5D,E,I,J). Similarly, neighbors’ average and maximum calcium parameters, describing frequency of transients (Supplementary Figure S5F,K), peaks (Supplementary Figure S5G,L) and activity period length (Supplementary Figure S5H,M) were higher around surviving vs. dying neurons. When combining observations on single cell and cluster level, by quantifying the probability of cell death given the firing state of the cell itself and its neighbors (Figure 5I, χ^2^ = 90.11, df = 3, *p* < 0.0001), the results showed that silent neurons with silent neighbors were more likely to die (29.5%), whereas active neurons with active neighbors had the best chance to survive (94.4%, i.e., 5.6% chance of cell death). Moreover, the survival rates both among silent neurons (70.5% with silent neighbors vs. 77.5% with active neighbors, χ^2^ = 5.17, df = 1, *p* < 0.025, post hoc comparison with Bonferroni corrected significance threshold) and active neurons (84.7 with silent neighbors vs. 94.4% with active neighbors, χ^2^ = 9.99, df = 1, *p* < 0.005, post hoc comparison with Bonferroni corrected significance threshold) were higher in the presence of active neighbors.

In conclusion, we show that the number of neighboring neurons is not associated with a preferred cell fate, but instead high frequency activity of neurons in close vicinity appears to be a protective factor against cell death. Active neighbors supported survival of silent and active neurons in immature networks, where connectivity still evolves even within clusters.

### 3.4 Modular Topology Forsters Survival Rates

**FIGURE 6:**
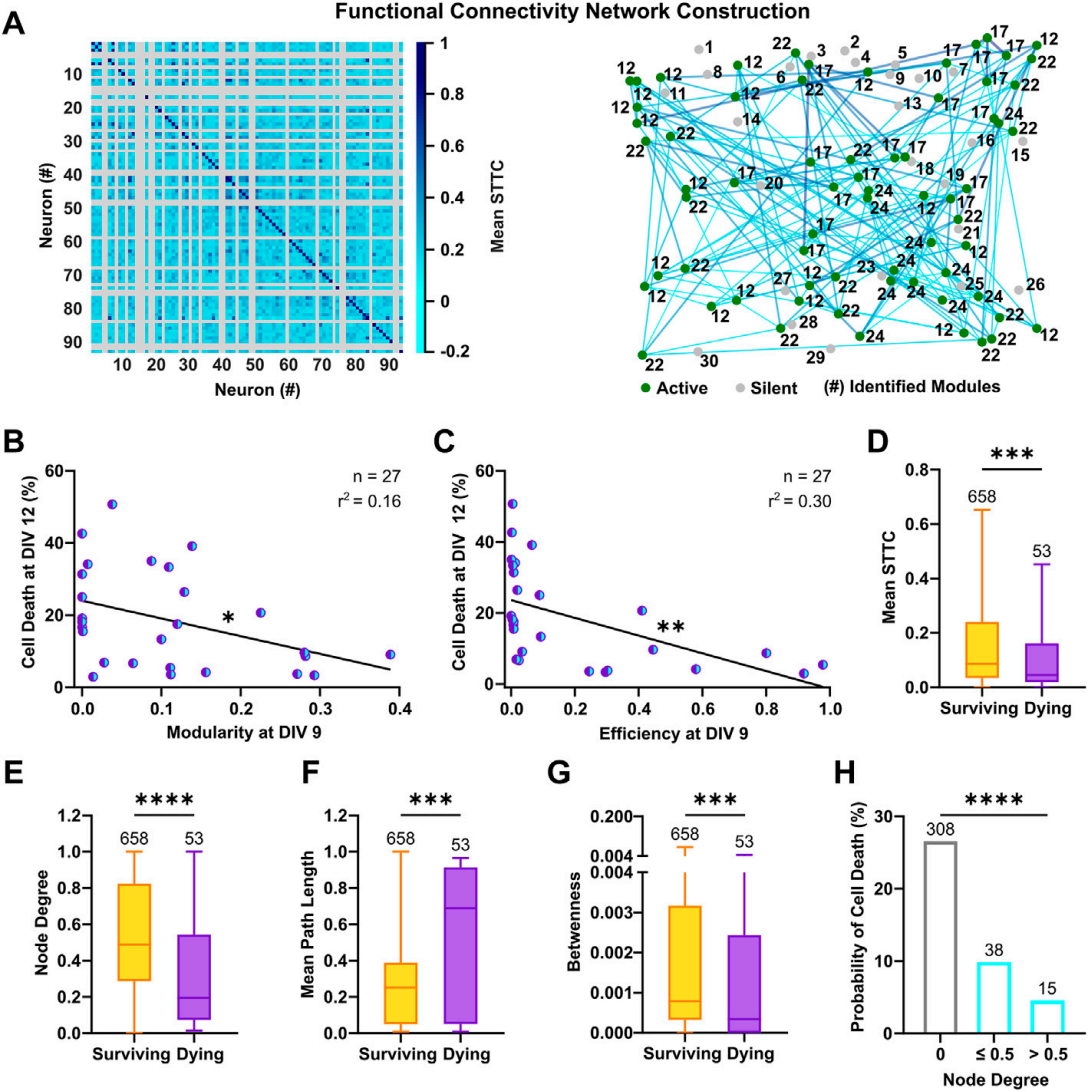
Functional connectivity fosters neuronal survival in culture. **(A)** Representative connectivity matrix (left) and network graph (right) of 93 neurons at DIV 9. Active (green) and silent neurons (gray) are shown in their physical positions with respective module numbers and representative functional connections (cyan-blue) are drawn between active neurons. **(B,C)** Modularity and efficiency of the network at DIV 9 were negatively correlated with subsequent cell death rates at DIV 12 (F test). **(D)** Surviving neurons showed a higher synchrony (mean STTC) with activity of other neurons in culture (Mann-Whitney test). **(E)** The number of connections (node degree) was higher in surviving neurons compared to neurons with a prospective dying fate (Mann-Whitney test). **(F)** Average path length at DIV 9 was longer for neurons, which died until DIV 12 (Mann-Whitney test). **(G)** Surviving neurons had a more central position (betweenness) within connections of others pairs of neurons (Mann-Whitney test). **(H)** The probability of cell death was lower in neurons with a higher node degree (Chi‐square test). Box plots with median and IQR and min–max whiskers or scatter plots with line indicating simple linear regression are shown. *p < 0.05, ***p < 0.001 and ****p < 0.0001.

The structural organization of cultured neurons in dense clusters sustains the formation of a stereotypical functional connectivity, which is fundamental for the generation of synchronous burst events across the network (Okujeni et al., 2017). To estimate the functional connectivity between neurons and ascertain its influence on neuronal survival, we calculated the pairwise synchrony between the reconstructed spike trains, by means of the spike time tiling coefficient (STTC). This measure is particularly suited to evaluate correlation between neuronal spikes in immature neurons, as it is not influenced by firing rates and common silent periods (Cutts and Eglen, 2014). By considering only positive correlations in the connectivity matrices, we built graphs in which neurons are linked by undirected connections and are organized in interconnected modules (Figure 6A). This division in strongly interconnected modules can be measured by an ad hoc index (Blondel et al., 2008), whose calculation revealed that networks with high modularity at an early developmental stage had lower cell death rates (Figure 6B, F_(1, 25)_ = 4.82, *p* < 0.05). In graph theory, networks with a modular configuration are topologically characterized by a high clustering coefficient and a low characteristic path length (for review, see Meunier et al., 2010), and have been shown to maximize information transfer. Accordingly, highly efficient networks, which favor communication between neurons (Achard and Bullmore, 2007), showed decreased rates of cell death (Figure 6C, F_(1, 25)_ = 10.46, *p* < 0.01) and the metrics descriptive of the underlying topology, clustering coefficient and characteristic path length, negatively correlated with cell death at DIV 12 (Supplementary Figure S6A,B). Within the framework of functional connectivity, an efficient information transfer across the network underlies co-activation between neurons, whereas silent cells are by definition disconnected from the network. We therefore compared functional properties of only the active cells based on their respective fate. At a single cell level, surviving neurons had an overall higher synchrony (Figure 6D, 8.7 × 10^−2^, 3.4 × 10^−2^–2.4 × 10^−1^ vs. 4.5 × 10^−2^, 1.9 × 10^−2^–1.6 × 10^−1^, *p* < 0.001, Mann-Whitney test) and a higher number of connections, as shown by the analysis of the node degree (Figure 6E, 0.49, 0.29–0.83 vs. 0.2, 0.07–0.54, *p* < 0.0001, Mann-Whitney test). Moreover, a shorter mean path length (Figure 6F, 0.25, 0.05–0.39 vs. 0.69, 0.05–0.91, *p* < 0.0001, Mann-Whitney test) in surviving neurons suggested a higher level of integration within the network, whereas the higher values of betweenness (Figure 6G, 7.9 × 10^−4^, 3.3 × 10^−4^–3.2 × 10^−3^ vs. 3.4 × 10^−4^, 0–2.4 × 10^−3^, *p* < 0.001, Mann-Whitney test) indicated a stronger ability to influence or being influenced by other network components. Despite these observations, we did not find indications of a higher number of hub neurons among the surviving subpopulation (Supplementary Figure S6C) and, in line with other studies (Schroeter et al., 2015), the small-worldness failed to capture significant differences across cultures (Supplementary Figure S6D). This suggests that specific measure of graph theory might have lower discriminative power at an early developmental stage or a lower correlation with programmed cell death. Similarly, clustering coefficient and local efficiency at single cell level and average betweenness at network level, did not show a significant correlation with cell death (Supplementary Figure S6E–G). To evaluate the influence of connectivity on neuronal survival, we next calculated the probability of cell death based on node degree. The results showed that neurons with lower connectivity display higher rates of cell death (Figure 6H, node degree 0, 26.6%, node degree ≤0.5, 9.9%, node degree >0.5, 4.6%, χ^2^ = 107.2, df = 2, *p* < 0.0001) and networks with higher connection density had lower cell death rates (Supplementary Figure S6H).

In conclusion, the analysis of functional connectivity revealed that the integration of neurons into strongly interconnected modules and highly efficient networks fosters neuronal survival. Furthermore, high synchrony and number of connections are beneficial for survival of individual neurons.

### 3.5 Activity Profiles are Predictive of Neuronal Survival

**FIGURE 7:**
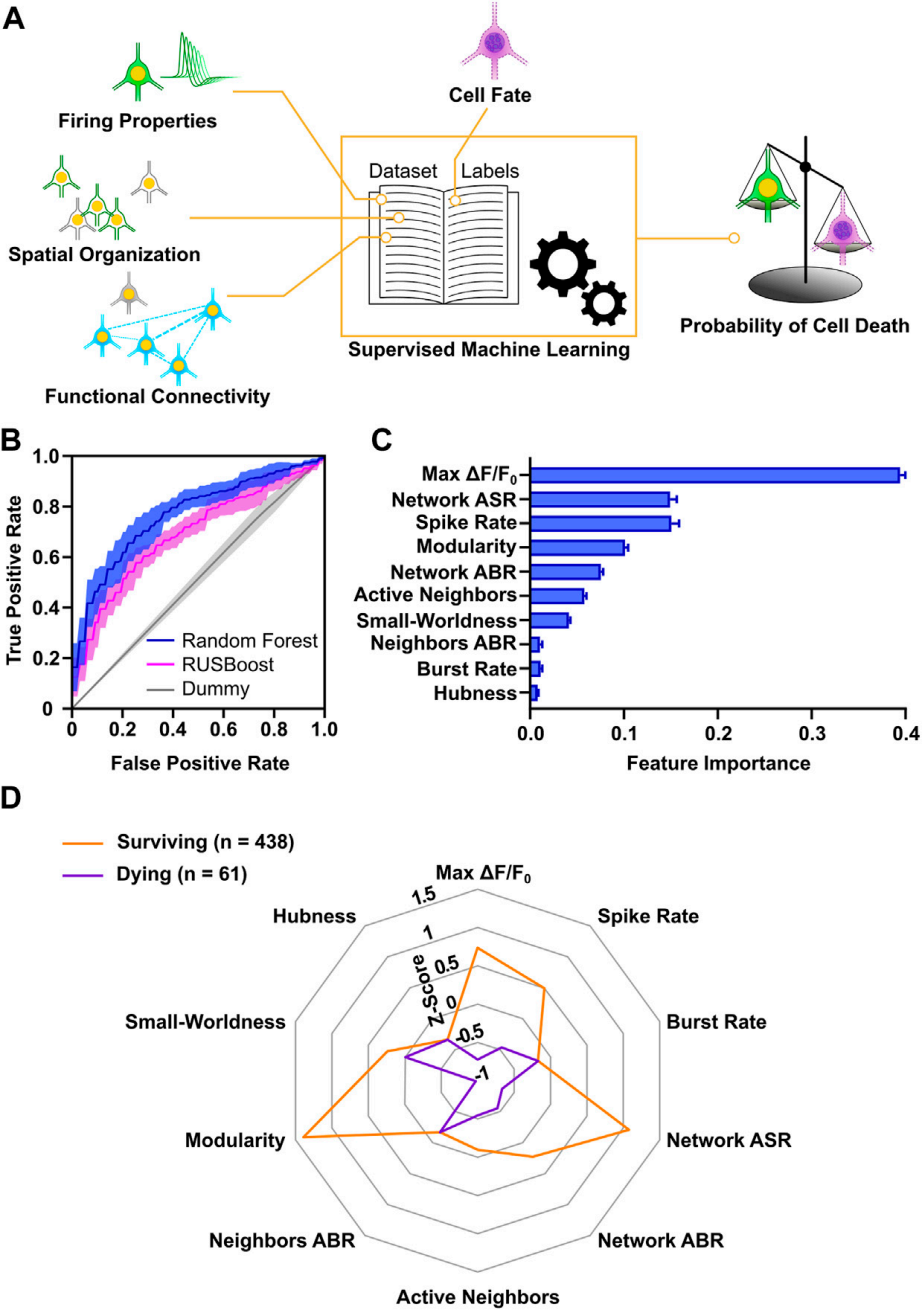
Survival of developing cortical neurons is predictable based on early activity profiles. **(A)** Schematic representation of workflow applied for machine learning approach. **(B)** Accuracy of Random Forest and RUSBoost algorithms exceeded Dummy prediction. AUROC curves (solid lines) indicate mean and envelope (semi-transparent area) SD of 10 fold cross-validation. **(C)** Relative informative weight of parameters used for the Random Forest classification. Bar chart represents mean values ± SD. **(D)** Radar chart shows comparison of activity profiles in surviving and dying neurons with an estimation accuracy of their cell fate greater than 80%. Modularity, network ASR, max ΔF/F_0_ and spike rate showed the greatest difference in Z-scores (Mann-Whitney test). Lines connect the median z-score value of each parameter. ASR, average spike rate; ABR, average burst rate.

The analysis of properties, at a single neuron, cluster and network level allowed us to gain insight on the conditions that promote neuronal survival in culture and characterize features of surviving and dying neurons. We next aimed at analyzing if the combination of these properties could provide a more in-depth description of activity profiles predictive of cell death or survival. For this purpose, we applied machine learning, which is a consolidated approach in neuroscience (for review, see Vu et al., 2018) and allows to identify nonlinear dependencies in multidimensional neurophysiological datasets (for review, see Cunningham and Yu, 2014). Moreover, this approach has been successfully used for prediction of spontaneous activity during early development on MEAs (Cabrera-Garcia et al., 2021). For each of the 1874 neurons, we included all parameters measured at DIV 9 describing neuronal firing, cluster spatial organization and network functional connectivity, and assigned a label according to its cell fate at DIV 12. By means of supervised learning, we classified the cells in the two different classes (surviving vs. dying) and computed the probability of survival or cell death (Figure 7A). The collected dataset amounted to a total of 46 variables, which as expectable displayed a strong degree of cross-correlation (Supplementary Figure S7A). To reduce the dimensionality of the dataset, 10 representative descriptors were retained from the groups of correlated variables (Supplementary Figure S7B). Label distribution was strongly biased towards the surviving population (1,513 vs. 361). Therefore, we selected two supervised classifiers, which typically have good performance on imbalanced datasets: the balanced Random Forest and the Random Undersampling Boosting (RUSBoost) classifiers (Lemaître et al., 2017). Both methods showed a good performance on the dataset, indicated by the area under the receiver operating characteristics curve (AUROC) values of 0.77 and 0.69, respectively (Figure 7B). As a comparative baseline, a Dummy classifier (Pedregosa et al., 2011) was used, which yielded an AUROC value of 0.51. In addition to the good performance in our dataset, the Random Forest classifier enabled to evaluate the relevance of each variable for the model. The resultant feature importance scores (Figure 7C) highlight that max ΔF/F_0_ carried the highest relative informative weight (about 40% of the total) and that a subset of 7 variables (max ΔF/F_0_, network average spike rate, spike rate, modularity, network average burst rate, active neighbors, small-worldness) accounted for more than 95% of the relative feature importance.

Next, in order to obtain a characterization of activity profiles distinguishing surviving from dying neurons, we selected all the neurons that were confidently predicted by the Random Forest classifier in each class (i.e., more than 80% probability of assignment to a class). For these 499 neurons with correctly predicted dying or surviving cell fate median z-scored values of the model variables were calculated (Figure 7D). Interestingly, the parameters showing the largest differences between surviving and dying cells were max ΔF/F_0_ (0.74, 0.05–1.75, vs. −0.72, −0.89–0.87 *p* < 0.0001, Mann-Whitney test), modularity (1.39, −0.04–1.49, vs. −0.98, −1.04 to −0.7, *p* < 0.0001, Mann-Whitney test) and network average spike rate (1.08, −0.25–1.82, vs. −0.66, −0.71 to −0.64, *p* < 0.0001, Mann-Whitney test). In agreement with the analysis of individual variables, this suggests that large somatic calcium increases in neurons, integration into highly interconnected neuronal modules, and high network firing rates are predictive of cell survival.

In conclusion, our results suggest that a subset of features at single cell and network level can be sufficient for identifying cell fate with a good confidence. In particular, parameters describing calcium dynamics, single neuron and network firing rates, and functional connectivity are a suitable and sufficient subset of activity features to predict survival of immature neurons.

## 4 Discussion

Spontaneous activity in the cortex regulates many neurodevelopmental processes, such as migration, integration into cortical circuits and programmed cell death of neurons (for review, see Luhmann et al., 2016; Martini et al., 2021; Warm et al., 2022). However, it remained unclear whether spontaneous activity patterns from network down to single neurons directly encode for survival of individual cortical neurons. Here, we investigated how intrinsic, local and network factors that contribute to the emergence of spontaneous activity during early development, affect survival of developing cortical neurons. The main results of our study can be summarized as follows: 1) The individual display of spontaneous calcium transients during early development strongly reduced the probability of cell death of cortical neurons; 2) Cortical neurons with high spontaneous firing rates were unlikely to undergo apoptosis and spike bursts were almost exclusively observed in surviving neurons; 3) Activity of close neighbors within neuronal clusters exerted a pro-survival effect; 4) A network functional topology characterized by high modularity at an early developmental stage fostered neuronal survival; 5) Large somatic calcium increases in neurons, integration into strongly interconnected neuronal modules, and high network firing rates were predictive of survival of developing cortical neurons. We conclude that survival of cortical neurons in developing neuronal networks is predictable based on spontaneous activity patterns.

Cortical neurons *in vitro* spontaneously exhibit activity, whose evolution recorded with microelectrode arrays resembles the perinatal progression of activity in the *in vivo* rodent cortex from decorrelated action potentials to synchronized network oscillations (Yang et al., 2009; Sun et al., 2010; for review, see Kilb et al., 2011). At the same time, primary cortical cultures provide the advantage of direct manipulation and observation of developing cortical neurons (for review, see Potter, 2001). In the present study, cortical neurons were genetically manipulated to simultaneously express the calcium indicator GCaMP6s and the nuclear tag nls-dTomato. This strategy allowed to longitudinally follow activity and apoptosis from network down to the single neuron level and to include in the analysis silent neurons, which are often underrepresented in electrophysiological studies (for review, see Shoham et al., 2006). The spontaneous display of calcium transients at an immature developmental stage, when network activity is dominated by sparse decorrelated action potentials strongly reduced the probability of death of individual cortical neurons. The absence of activity, instead, lowered the likelihood of neuronal survival. Conversely, a general decrease in cell death rates was observed in the late phase of the second week *in vitro*, when neuronal activity transitions to synchronous burst activity, driving almost all neurons in the field of view to display calcium transients. During initial phases of development in culture, surviving neurons not only displayed higher rates, but also higher amplitudes of calcium transients. Intracellular calcium has been long known to support neuronal survival (for review, see Franklin and Johnson, 1992; Ghosh and Greenberg, 1995) and for sustained calcium elevations to occur, voltage‐gated Ca^2+^ channels (VGCC) exert a primary role (for review, see Moody and Bosma, 2005). Especially in immature neurons, calcium currents contribute to the generation of slow action potentials (Luhmann et al., 2000) and uncorrelated TTX-resistant calcium transients have been observed (Corlew et al., 2004). In line with these evidences, we found that the mere application of TTX only marginally affected neuronal survival, whereas combined application with the VGCC blocker isradipine resulted in full blockade of calcium transients and strongly reduced neuronal viability at an early stage in network development.

In the rodent cortex, early network activity during postnatal development is characterized by high frequency oscillations in the beta and gamma range (Yang et al., 2009; Colonnese et al., 2010; Minlebaev et al., 2011) and analogous patterns are also described in cortical *in vitro* models (Dupont et al., 2005; Gireesh and Plenz, 2008). On the population level, higher spectral power in this range has been associated with higher survival rates (Blanquie et al., 2017a) and disruption of early activity patterns leads to abnormal cell death rates in the cortex (Duan et al., 2020; Bitzenhofer et al., 2021). In the present study, the simultaneous measurement of neuronal activity with electrophysiology and calcium imaging enabled the parameter tuning of a spike inference algorithm and the accurate reconstruction of spiking activity in a large population of developing cortical neurons. Although slow decay kinetic and nonlinearity of the calcium indicator GCaMP6s imposes a low-pass filter on neuronal activity (Wei Z et al., 2020) and thus limited the maximal frequency range of the inferred spikes, we show that higher spike rates and burst behavior were almost exclusive properties of surviving neurons. Thus, our findings underline how high frequency firing determines lower probability of cell death also at a single cell level and corroborates high firing rate and bursting behavior as hallmark features of surviving neurons, previously observed on a population level (Golbs et al., 2011).

During early development, calcium transients and burst activity contribute to the proper shaping of cortical circuits (Golbs et al., 2011; Murase et al., 2011; for review, see Kirkby et al., 2013). In this regard, neurons in culture self-organize in clusters (Robert et al., 2012), and their aggregation during the first days *in vitro* is influenced by activity (Jeong et al., 2009; Shein Idelson et al., 2010). Concurrently with the formation of these clusters, neurons start forming synaptic connections, which progressively engage more neurons in synchronous firing (Opitz et al., 2002; Soriano et al., 2008). Within this context, we observed that both silent and active surviving neurons were surrounded by a higher number of active neighbors and by neighbors with a higher firing frequency. This suggests that cortical neurons surrounded by highly active neurons are more likely to be integrated into local assemblies and to participate into emerging correlated synchronous activity that ultimately support their survival. Along this line, tonic or high frequency firing neurons might attract nearby neurons into local clusters (Feinerman et al., 2007) and concomitantly test their aptness to integrate into forming circuits. In line with the higher probability of death among the silent subpopulation, neurons that fail to respond within a short developmental window might be more likely to activate or sustain preset apoptotic programs.

On the network level, the clustered conformation of neuronal cultures reflect the emergence of a modular functional topology that is evident at different scales in the brain and has evolved to maximize efficiency of information transfer (for review, see Bullmore and Sporns, 2009). This functional organization makes neuronal networks more robust against loss of individual nodes (Achard et al., 2006) and ensures the segregation of different functional cortical areas, while allowing the generation of coherent perceptual and cognitive states across the brain (for review, see Sporns et al., 2000). Here, we show that an early achievement of a modular topology reduced apoptotic rates in culture. Such network configuration is highly efficient for information transfer and promotes the emergence of synchronous activity (Okujeni et al., 2017). Accordingly, our results show that networks with high efficiency displayed lower rates of cell death and surviving neurons showed an overall higher synchrony than dying ones. In this view, synchronous oscillations efficiently transmitted across the network might reinforce connectivity (Mohajerani et al., 2007). Indeed surviving neurons had an overall higher node degree and shorter path length. Since neither the density, nor the spatial distribution of neurons in culture were correlated with cell death rates, variability in functional topology across network at the early stages of development might reflect the heterogeneity of cortical neuronal populations (Voigt et al., 2001) or a certain degree of randomness in their wiring.

Our data provide an extensive characterization of factors influencing neuronal survival from different scales of spontaneous activity. Many of the analyzed activity parameters showed large variability and partially overlapping distributions between surviving and dying neurons. This likely reflects differences in neuronal subtypes or maturational stages across individual neurons and indicates the multiplicity of factors regulating cell death. To identify these and reveal predictive features of survival or cell death of individual neurons, we applied machine learning, which use as diagnostic and prognostic tool is recently emerging to assess brain development based on EEG recordings of preterm infants (Wei L et al., 2020; for review, see Tataranno et al., 2021) and for early prediction of spontaneous activity in cortical networks *in vitro* (Cabrera-Garcia et al., 2021). The overall good performance of the applied classifiers demonstrated how the survival fate of immature neurons is predictable based on activity features from single neuron, cluster and network level. The use of the standard algorithm Random Forest (for review, see Chicco, 2017) enabled a direct interpretation of the relevance of the features and indicated that even a subset of seven descriptors is sufficient for correct estimation of cell fate. Moreover, the comparison of activity profiles between neurons accurately predicted to survive or to die demonstrated the importance of calcium dynamics, network modular organization and firing rates for neuronal survival.

In conclusion, our results suggest that high frequency spiking, whose information load is efficiently propagated in networks with a high modular topology (for review, see Meunier et al., 2010), translates into sustained calcium rises in single neurons and finally constrains their apoptotic loss. This implies that survival of individual neurons within developing cortical networks is supported by the engagement into synchronous high frequency firing, but also denotes that through the activity-dependent downregulation of cell death rates, cortical circuits reach a structural stability that is beneficial for information processing in mature networks (for review, see Robinson et al., 2009). In this way, spontaneous activity provides a regulatory mechanism that, interacting with predefined genetic programs and environmental cues, can control cellular composition and refine local circuitry across different cortical regions and developmental time points *in vivo* (for review, see Spitzer, 2006).

Despite its reduced complexity, the cortical culture model recapitulated fundamental structural and functional hallmarks of neuronal network development, which allowed us to systematically assess the relevance of activity features from the single neuron to the network level for the survival of developing cortical neurons. We show that in individual neurons spontaneous activity is a strong positive prognostic factor for their survival and that the combination of few parameters from the network down to the single neuron level is sufficient to predict cell fate during development. This study can thus form the basis of interventional experiments and our findings could prompt future investigation on how specific activity patterns translate in pro-survival programs.

## Data Availability

The raw data supporting the conclusion of this article will be made available by the authors, without undue reservation.
