# Supplementary material for: Spontaneous Activity Predicts Survival of Developing Cortical Neurons
Source: Front Cell Dev Biol. 2022 Aug 10;10:937761. doi: 10.3389/fcell.2022.937761 (PMC9399774; doi:10.3389/fcell.2022.937761)
Supplement: Supplementary file 2 [file DataSheet1.pdf]

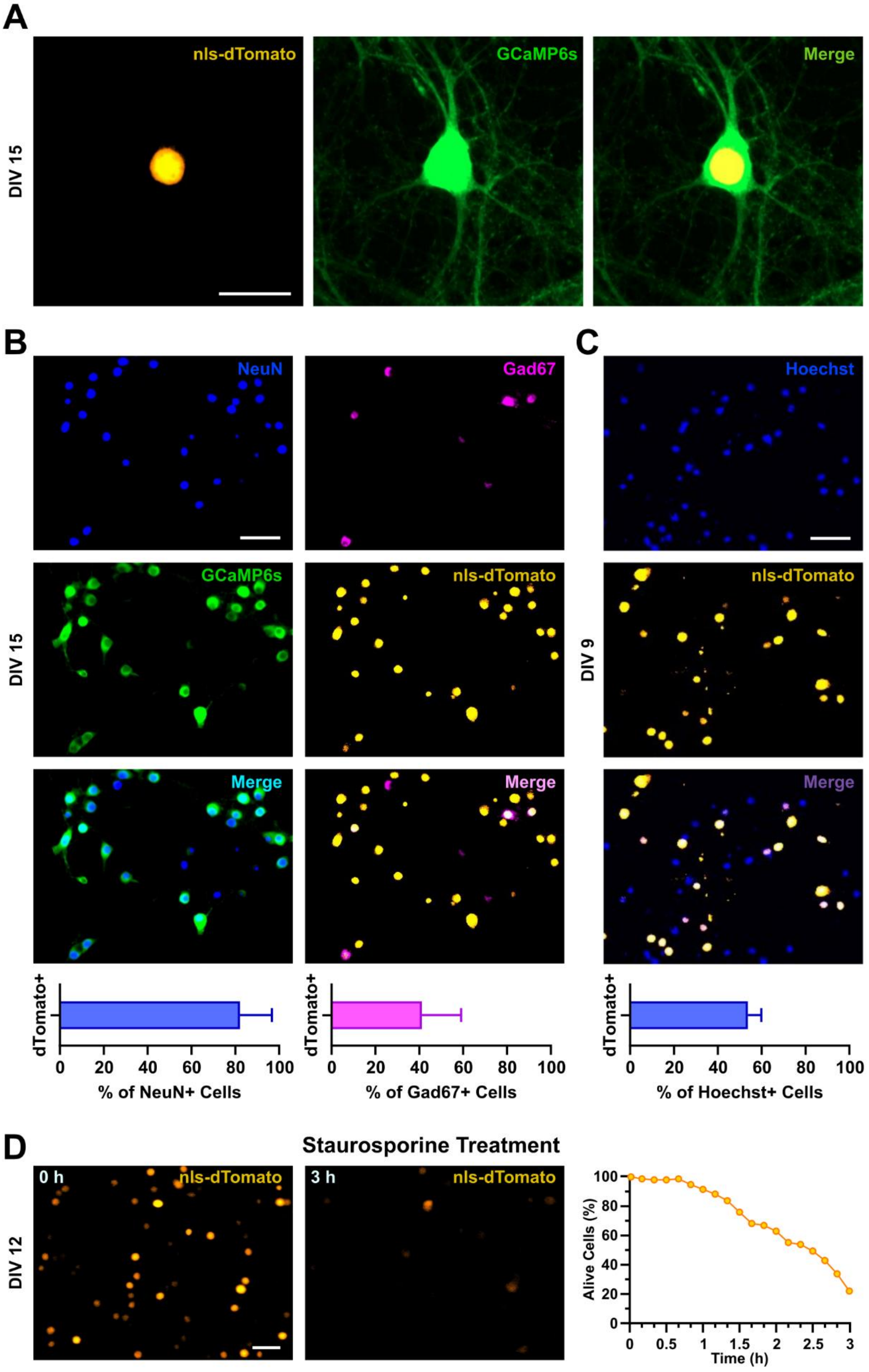

SUPPLEMENTARY FIGURE S1 | Viral strategy allows widespread targeting of neurons and monitoring of cell fate. **(A)** Confocal maximal z-projection showing the nuclear localization of nls-dTomato (left), the somatic signal of GCaMP6s (center) and their merge signals (right) in a cortical neuron after immunocytochemistry. **(B)** Representative immunolabeling of cortical neurons against NeuN (blue), GCaMP6s (green) and merge of their signals (cyan) on the left, whereas stainings for Gad67 (magenta), nls-dTomato (yellow), and their merge (pink) are shown on the right. Quantification of transduction rates indicated that 82.1% ± 14.6% (n = 25) of neurons in culture were targeted, but lower expression of transgenes was present in Gad67+ cells (41.2% ± 18%, n = 12). **(C)** At early stage, 53.7% ± 6.1 (n = 12) of alive neurons, detected with Hoechst staining (blue), showed expression of nls-dTomato (yellow). **(D)** Disappearance of nls-dTomato signal (yellow) allowed rapid detection of cell death. Upon staurosporine treatment, the number of alive neurons strongly decreased over the following 3 hours. Image scale bars represent 20 μm **(A)** and 50 μm **(B–D)**.

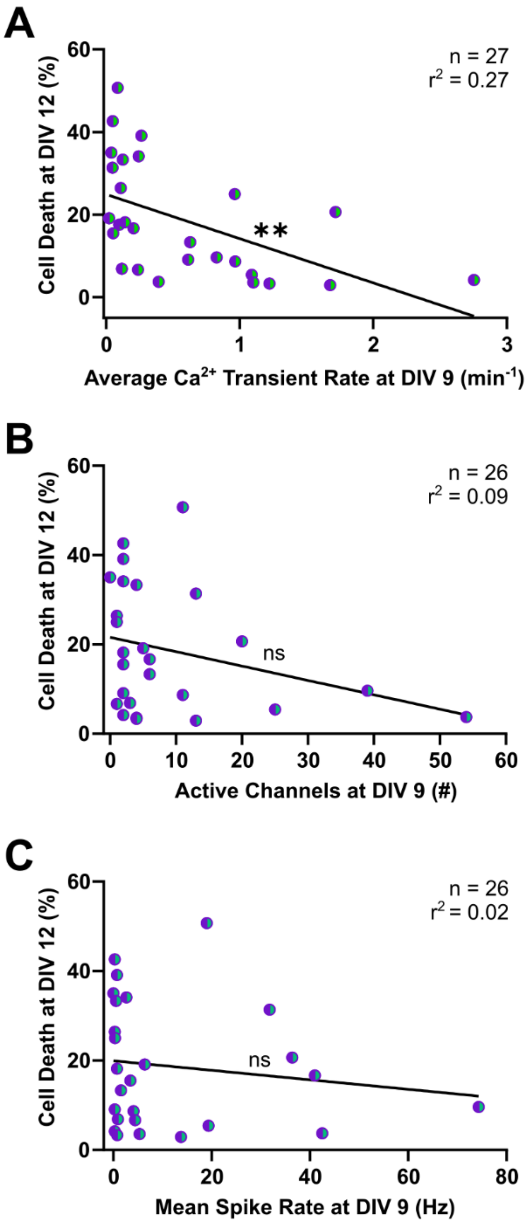

SUPPLEMENTARY FIGURE S2 | MEA network parameters show weak association with cell death rates. **(A)** Average calcium transient rate negatively correlated with cell death at DIV 12 ($F_{(1, 25)}$ = 9.45). **(B,C)** Common MEA parameters, such as number of active channels ($F_{(1, 24)}$ = 2.27) and mean spike rate ($F_{(1, 24)}$ = 0.49), did not show significant correlation with neuronal apoptosis in culture. ***p* < 0.01.

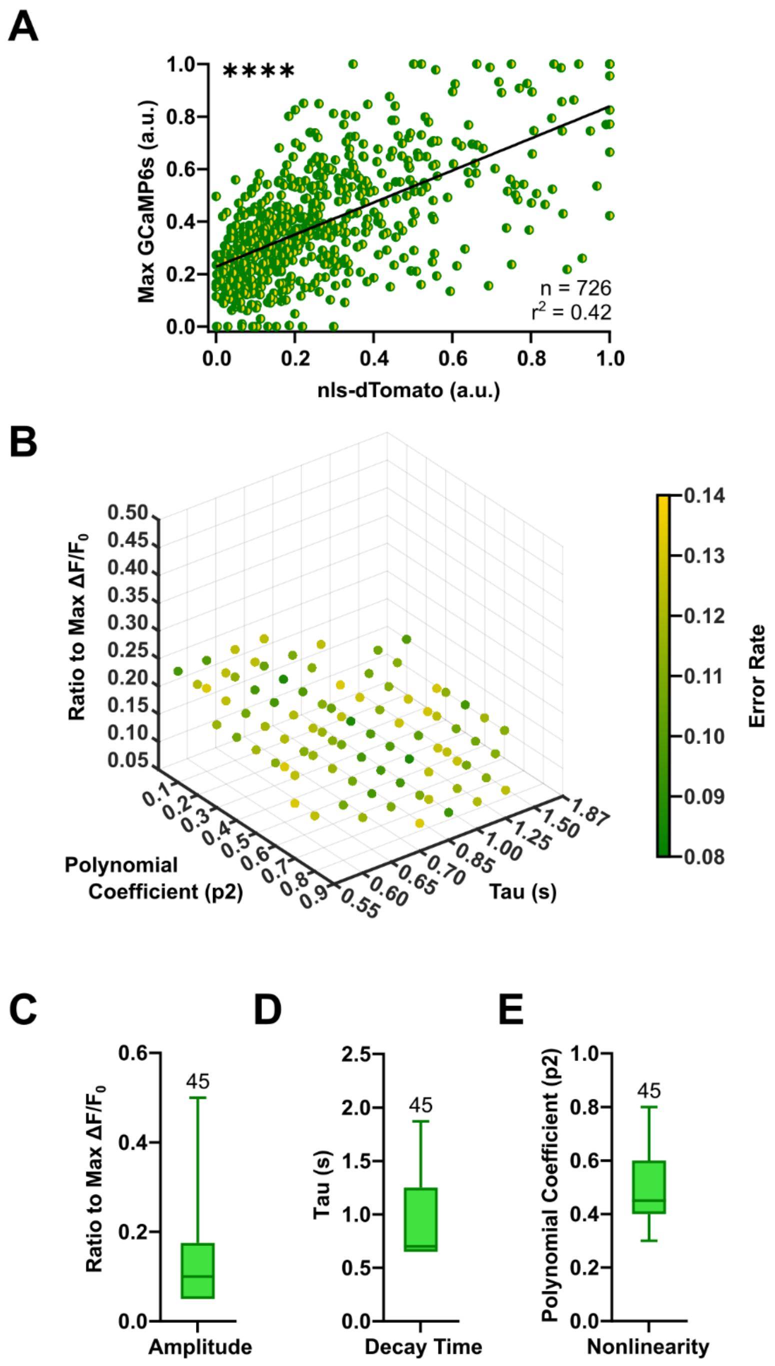

SUPPLEMENTARY FIGURE S3 | Parameter tuning of MLspike algorithm. **(A)** Correlation between the nuclear nls-dTomato signal and the somatic GCaMP6s maximal intensity over the recording period at DIV 9. Fluorescence signals of active neurons were pooled after normalization within each culture ($F_{(1, 724)}$ = 514). Line indicates simple linear regression. **(B)** Representative grid search over different values of the model parameters used by the MLspike algorithm. 3D scatter plot shows best candidate values based on the 10% lowest error rates between the recorded and the reconstructed spike train of one neuron. **(C–E)** Optimal values of the model parameters were chosen as the median across 45 neurons used for fitting the model (ratio to max $\Delta F/F_0$ = 0.1, 0.05–0.18; tau = 0.7, 0.65–1.25; p2 = 0.45, 0.4–0.6). Box plots with median and IQR and min–max whiskers are shown. ****p < 0.0001.

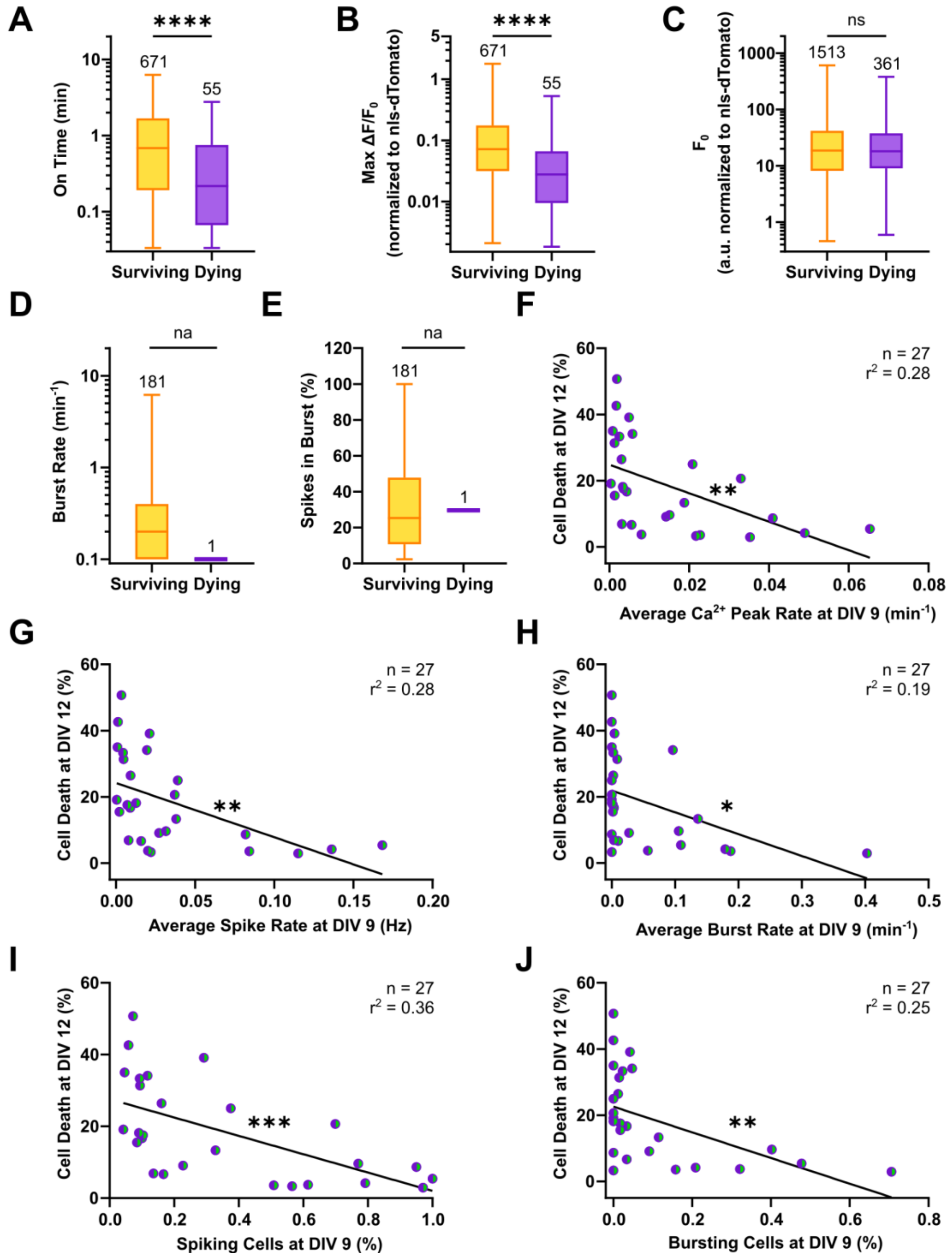

SUPPLEMENTARY FIGURE S4 | Neuronal firing properties are independent of GCaMP6s expression and correlate with apoptosis at network level. **(A)** Surviving neurons were active for longer periods during the recording time, compared to neurons that died until DIV 12 (0.68, 0.19–1.68 vs. 0.21, 0.07–0.75 minutes, Mann-Whitney test). **(B)** Normalization of the max $\Delta F/F_0$ to the fluorescence intensity of nls-dTomato revealed that the higher amplitude of calcium transients in active neurons with a surviving vs. a dying fate did not depend on the GCaMP6s expression levels (7.2×10^{-2}, 3.2×10^{-2}–1.8×10^{-1} vs. 2.8×10^{-2}, 9.4×10^{-3}–6.6×10^{-2}, Mann-Whitney test). **(C)** Normalized baseline fluorescence F_0 (a.u.), in both active and silent neurons, did not differ between surviving and dying neurons (18.6, 8.2–41.7 vs. 18.2, 9–37.6, Mann-Whitney test). **(D,E)** Quantification of burst parameters: surviving neurons showed 0.2 (0.1–0.4) burst per minute and a proportion of spikes in burst of 25.3% (10.8–47.8); only in 1 dying neuron, bursts could be detected with a rate of 0.1 per minute and a proportion of spikes in burst of 29.7%. **(F–J)** Average network parameters computed after characterization of single neuron firing properties confirmed negative correlation between activity and subsequent cell death rates. The corresponding F test with degrees of freedom (1, 25) yielded the following F values: average calcium peak rate = 9.84; average spike rate = 9.66, average burst rate = 5.96; spiking cells = 14.22; bursting cells = 8.2. Box plots with median and IQR and min–max whiskers or scatter plots with line indicating simple linear regression are shown. **p* < 0.05, ***p* < 0.01, ****p* < 0.001, *****p* < 0.0001.

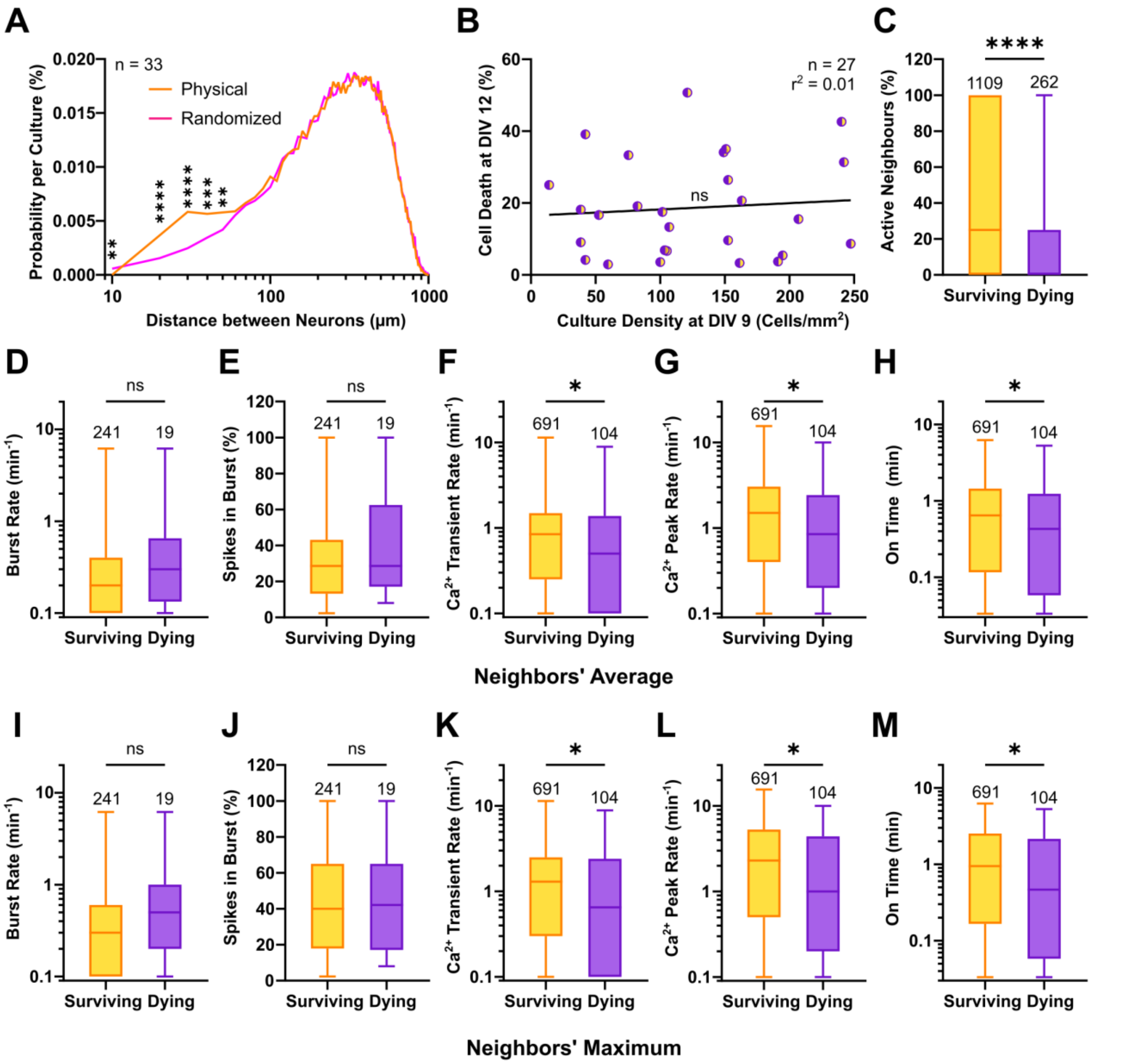

SUPPLEMENTARY FIGURE S5 | Pro-survival effect of active neighbors depends on their firing properties and not on culture density. **(A)** Compared to randomized coordinates, neurons in the field of view tended to distribute within a 50 µm distance from each other at a higher probability (two-way ANOVA test with Geisser-Greenhouse correction followed by Šidák's multiple comparisons test). Lines show mean values. **(B)** Variability of cell density within the adopted culturing conditions did not influence cell death rate ($F_{(1,25)} = 0.19$, $p = 0.67$). Line indicates simple linear regression. **(C)** Compared to neurons that survived until DIV 12, dying neurons had a lower percentage of active neurons in their vicinity (25, 0–100, vs. 0, 0–25, Mann-Whitney test). Average **(D–H)** and maximal **(I–M)** values of firing properties across active neighbors. Surviving neurons were surrounded by neighbors with higher levels of spontaneous activity, compared to neurons that died until DIV 12. In particular, average **(F)** and maximum **(K)** calcium transient rates (min^{-1}) were respectively 0.84, 0.25–1.48 vs. 0.5, 0.1–1.38 and 1.3, 0.3–2.5 vs. 0.65, 0.1–2.4; average **(G)** and maximum **(L)** calcium peak rates (min^{-1}) were respectively 1.5, 0.4–3.1 vs. 0.85, 0.2–2.43 and 2.3, 0.5–5.3 vs. 1, 0.2–4.4; average **(H)** and maximum **(M)** on time (min) were respectively 0.65, 0.12–1.45 vs. 0.43, 0.06–1.24 and 0.95, 0.17–2.53 vs. 0.47, 0.06–2.15. However, the properties of bursting neighbors close to surviving or dying neurons did not substantially differ. In particular, average **(D)** and maximum **(I)** burst rates (min^{-1}) were respectively 0.2, 0.1–0.4 vs. 0.3, 0.13–0.65 and 0.3, 0.1–0.6 vs. 0.5, 0.2–1; average **(E)** and maximum **(J)** spikes in burst (%) were respectively 28.7, 13.3–43.1 vs. 28.7, 17.1–62.5 and 40, 17.9–65 vs. 42.1, 17,1–65. Mann-Whitney test was applied to **(D–M)**. Box plots with median and IQR and min–max whiskers are shown. $*p < 0.05$, $**p < 0.01$, $***p < 0.001$, $****p < 0.0001$.

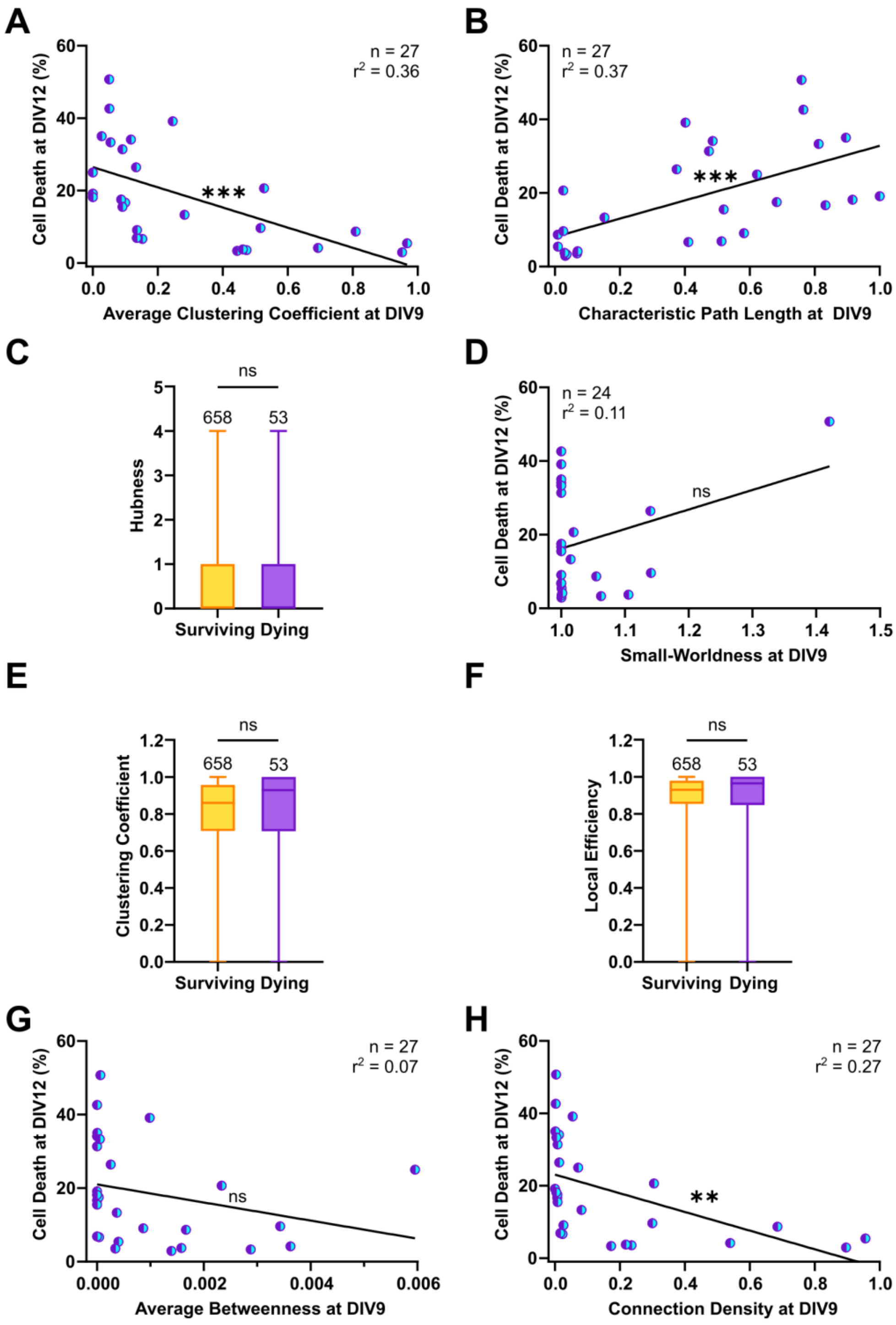

SUPPLEMENTARY FIGURE S6 | Higher survival rates are associated to network modular topology, but not to small-worldness. **(A,B)** Networks with high average clustering coefficient ($F_{(1, 25)}$ = 14.09) and low characteristic path length ($F_{(1, 25)}$ = 14.46) showed the lowest cell death rates. **(C)** Distribution of hub neurons did not differ between surviving and dying populations (0, 0–1 vs. 0, 0–1, p = 0.17, Mann-Whitney test). **(D)** Although some networks displayed small-world properties, its index did not correlate with survival rate ($F_{(1, 22)}$ = 2.81). Networks with clustering coefficient equal to 0 (n = 3) precluded estimation of the small-worldness index and were omitted from the graph. **(E,F)** Additional properties such as clustering coefficient (0.86, 0.71–0.96 vs. 0.93, 0.71–1, p = 0.08, Mann-Whitney test) and local efficiency did not differ between surviving and dying neurons (0.93, 0.85–0.98 vs. 0.96, 0.85–1, p = 0.1, Mann-Whitney test). **(G)** Average betweenness was not correlated with cell death rate ($F_{(1, 25)}$ = 1.91, p = 0.18). **(H)** High connection density correlated with high survival rate ($F_{(1, 25)}$ = 9.30). Box plots with median and IQR and min–max whiskers or scatter plots with line indicating simple linear regression are shown. $^{**}p < 0.01$, $^{***}p < 0.001$.

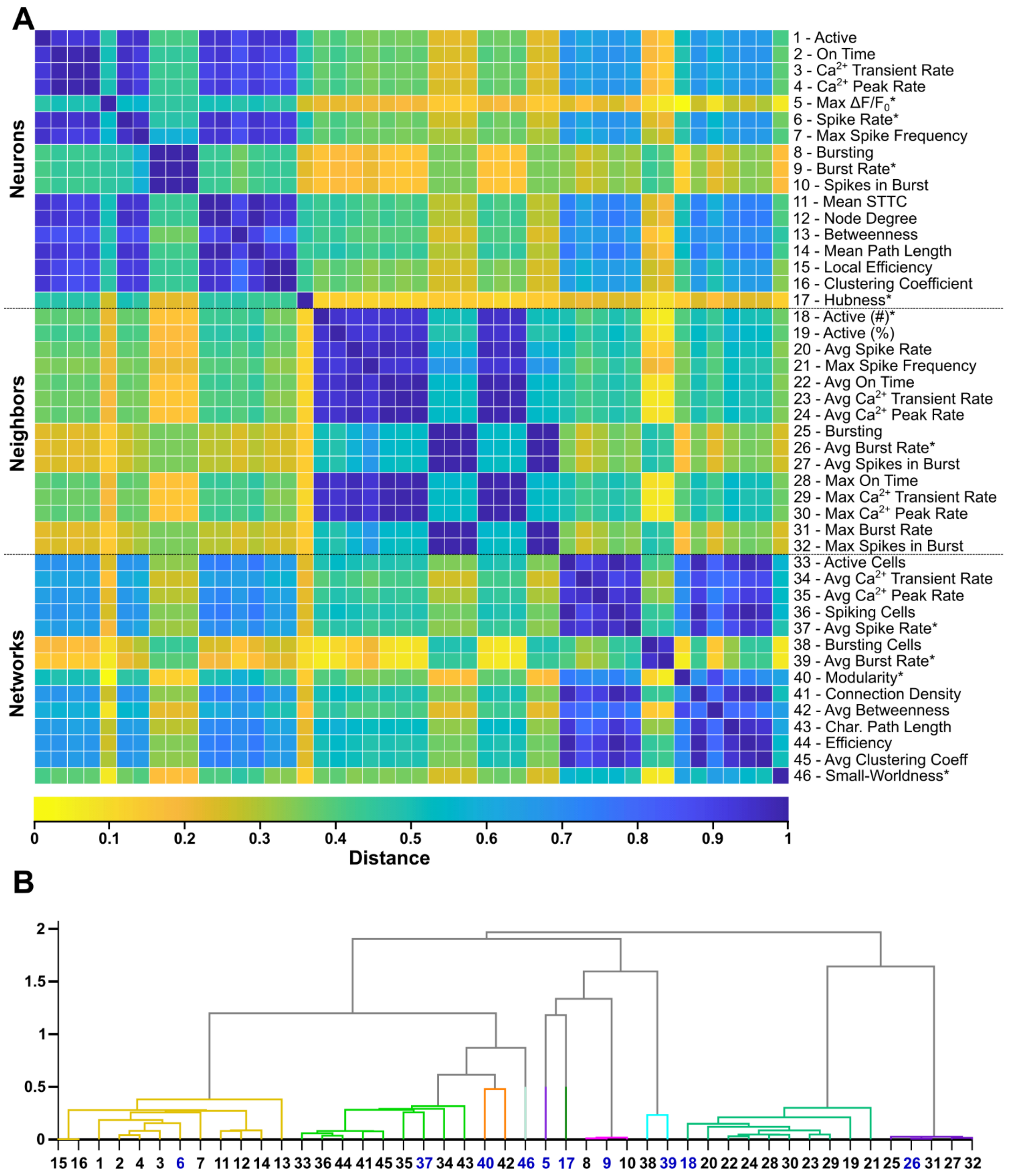

SUPPLEMENTARY FIGURE S7 | Feature selection for machine learning. **(A)** Multicollinearity of spontaneous activity features depicted using a distance matrix based on Spearman's rank correlation coefficient. Dashed lines separate features in 3 groups, based on their level: neuron, neighbor and network. * indicates variables used for prediction of cell fate. **(B)** Hierarchical clustering of the features based on the shortest Euclidean distance. A threshold of 0.5 was selected upon visual inspection and used to select a single feature for each cluster (indicated by the blue labels). The height in the dendrogram represents how distant are the nodes taking part in the connection.
